# AMPK regulates germline stem cell quiescence and integrity through an endogenous small RNA pathway

**DOI:** 10.1371/journal.pbio.3000309

**Published:** 2019-06-05

**Authors:** Pratik Kadekar, Richard Roy

**Affiliations:** Department of Biology, McGill University, Montreal, Quebec, Canada; Institute of Molecular Biology, Germany

## Abstract

During suboptimal growth conditions, *Caenorhabditis elegans* larvae undergo a global developmental arrest called “dauer.” During this stage, the germline stem cells (GSCs) become quiescent in an AMP-activated Protein Kinase (AMPK)-dependent manner, and in the absence of AMPK, the GSCs overproliferate and lose their reproductive capacity, leading to sterility when mutant animals resume normal growth. These defects correlate with the altered abundance and distribution of a number of chromatin modifications, all of which can be corrected by disabling components of the endogenous small RNA pathway, suggesting that AMPK regulates germ cell integrity by targeting an RNA interference (RNAi)-like pathway during dauer. The expression of AMPK in somatic cells restores all the germline defects, potentially through the transmission of small RNAs. Our findings place AMPK at a pivotal position linking energy stress detected in the soma to a consequent endogenous small RNA–mediated adaptation in germline gene expression, thereby challenging the “permeability" of the Weismann barrier.

## Abstract 2

When energy stress triggers dauer formation in nematodes, the cellular energy sensor AMPK genetically interacts with a small RNA pathway to mediate appropriate chromatin modifications and gene expression to regulate germline stem cell quiescence and integrity.

## Introduction

It is becoming more widely accepted that life history can affect developmental and behavioral outcomes in either a temporary or often a more permanent manner. These modifications can occur downstream of a broad spectrum of environmental factors, including temperature, light, resource availability, population density, and even the presence of predators, all of which can influence gene expression, often with dramatic phenotypic consequences [1, 2]. Furthermore, these consequences are not restricted to the generation that experienced the event, but rather, they can be transmitted to subsequent generations [3].

Studies have shown that the molecular record of these events is encoded in the form of epigenetic changes associated with histone modifications, DNA methylation and/or base modification, or alterations in the small RNA repertoire [4]. Because the transmission of these molecular memories can span one or several generations, these modifications must impinge in some way upon the germ line, thus providing an adaptive phenotypic change in the unexposed future generations [5–8]. These epigenetic modifications in the germ cells can have a significant impact on successive generations, yet the molecular mechanisms through which “experience" is transduced to the genome across several generations remain ill-defined.

*C*. *elegans* has been used successfully to demonstrate how environmental cues can modulate epigenetic change and behavior [9]. Furthermore, a subset of these modifications and associated traits can be transmitted to subsequent generations in a manner dependent on small heritable RNAs [10]. Recently, it was shown that acute starvation at the L1 larval stage leads to the generation of small RNA species that are inherited for at least three generations. This heritable pool of RNAs could reflect the adaptive change in the expression of genes involved in nutrition and metabolism [7, 11].

In addition to the L1 stage, later in development, larvae can execute an alternative developmental program to enhance survival and fitness in response to overcrowding or suboptimal growth conditions. During this diapause-like state, called dauer, they undergo a global, genome-wide adjustment of chromatin modifications that is accompanied by a significant change in gene expression when compared to the animals that never transited through this stage [12]. These changes in the abundance and distribution of chromatin marks likely contribute to the adaptive adjustment in gene expression that accompanies this developmental turnout and is most probably dependent on the expression of specific endogenous small RNAs [12, 13]. Currently, it is still unclear how the physiological stress associated with the dauer stage might impact the population of small RNAs and, in transgenerational contexts, how these changes are transmitted across generations, particularly in light of the erasure of histone marks that normally takes place during each cycle of embryogenesis [14].

Global cell cycle arrest is one of several distinctive features of *C*. *elegans* dauer larvae [15]. Upon entry into the dauer stage, the germline stem cell (GSC) divisions begin to slow to finally establish a state of quiescence, which they maintain until they recover from dauer and resume normal development [16]. Despite potentially long periods in this diapause stage, this cell cycle/developmental quiescence has no impact on their reproductive fitness [12]. Maintaining a quiescent state in response to energetic stress may be favorable for survival, presumably because it reduces energy consumption during a period when resources are limited [17, 18]. The activity of the cellular energy sensor AMP-activated Protein Kinase (AMPK) and its upstream kinase Liver Kinase B1 (LKB1)/PARtitioning defective-4 (PAR-4), as well as the activity of tumor suppressor Phosphatase and TENsin homologue (PTEN)/DAuer Formation abnormal (DAF)-18, are all independently required to establish the quiescent state of the GSCs in response to dauer signaling [19]. Upon activation due to decreased energy levels, AMPK enhances ATP production by promoting catabolic pathways while conserving ATP by inhibiting anabolic pathways. In doing so, AMPK regulates several aspects of cellular function, including cell growth, metabolic reprogramming, autophagy, cell polarity, and chromatin/epigenetic modification [5, 20–22].

The temperature-sensitive hypomorphic allele of the unique *C*. *elegans* insulin-like receptor (*daf-2(e1370)*) causes constitutive dauer formation when these mutants are reared at the restrictive temperature (25 °C) and initiates recovery from dauer at permissive temperature (15 °C) [23, 24]. Using this invaluable genetic tool, we found that AMPK activity was not only required to block GSC proliferation but also to maintain GSC integrity throughout the dauer stage to ensure reproductive success following recovery to replete conditions. In the absence of AMPK, several chromatin modifications become misregulated, resulting in inappropriate gene expression that has a detrimental effect on reproductive fitness following exit from the dauer stage. Using genetic analysis, we reveal the importance of the endogenous small RNA pathway and its regulation by AMPK. Moreover, this pathway acts at least partially in a cell nonautonomous manner to adjust the GSC-specific chromatin landscape, potentially in favor of an adaptive gene expression program fine-tuned toward maintaining germ cell integrity during the long-term energy stress typical of the dauer stage.

## Results

### Defects in the dauer germ line result in post-dauer sterility in AMPK mutants

In *C*. *elegans*, the decision to execute dauer development is regulated by three independent signaling pathways that converge on a nuclear hormone receptor to ultimately affect multiple developmental and physiological processes [25]. Many of these processes involve measures to conserve energy for the duration of the diapause, which are mediated through a significant metabolic adjustment that occurs downstream of all these signaling pathways [26].

To conserve energy while also ensuring that GSCs do not replicate during this period when key cellular building blocks may be limited, the *C*. *elegans* orthologues of LKB1 (*par-4*) and the regulatory and catalytic components of AMPK cooperate to establish cell cycle and developmental quiescence in the GSCs. Animals that lack both the catalytic subunits AMP-activated Protein Kinase (*aak*)*-1* and *aak-2* and thus all AMPK activity (henceforth referred as *aak(0)*) undergo pronounced germline hyperplasia because of supernumerary cell divisions that occur prior to dauer entry [13]. It is unclear, however, whether these extra cells remain reproductively competent to yield functional gametes.

**Fig 1:**
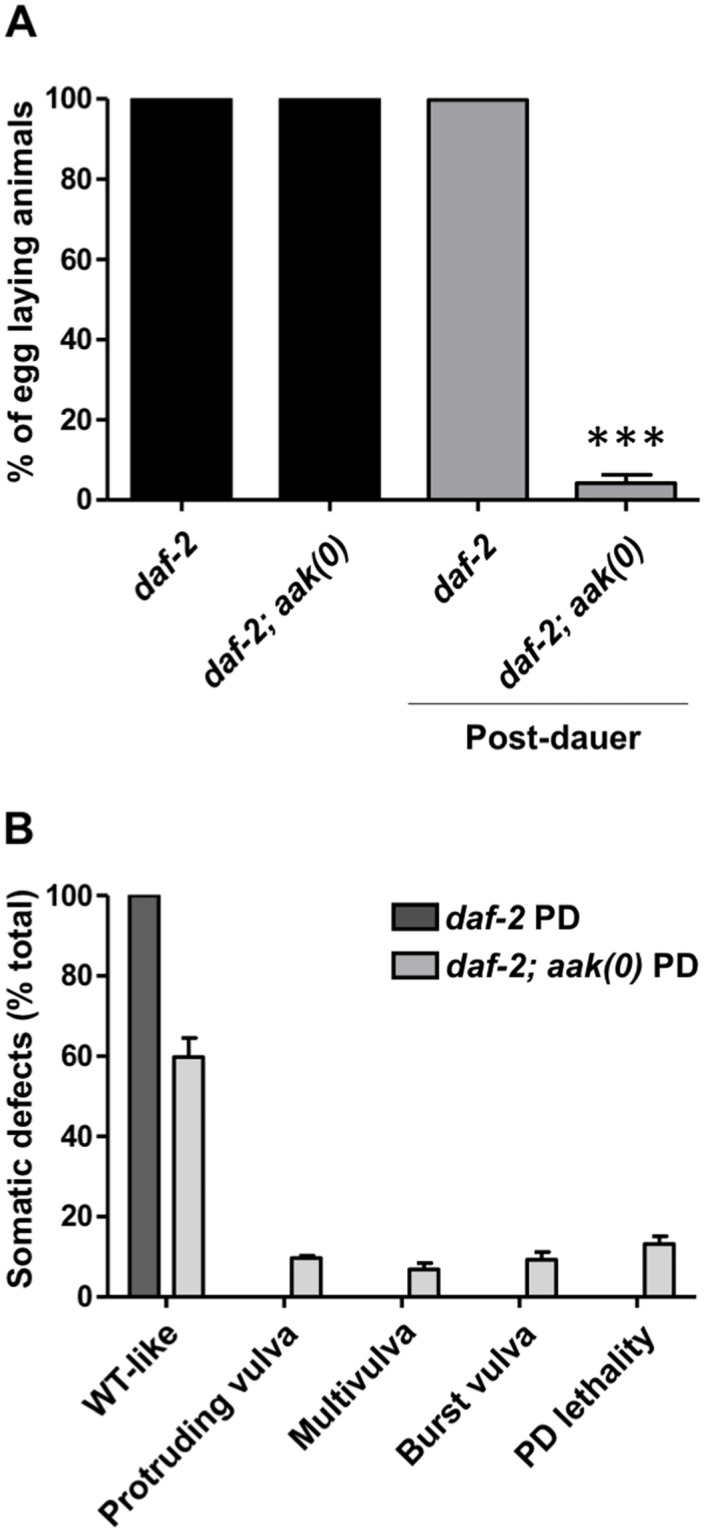
PD *aak(0)* adults exhibit vulval defects and highly penetrant sterility. (A) All adult animals that laid eggs were considered as fertile. Both *daf-2* and *daf-2; aak(0)* animals cultivated under permissive conditions showed no fertility defects compared to the wild type. To assess the fertility of the PD adults, animals were maintained in the dauer stage for 24 h, after which they were switched to permissive temperature to resume reproductive development (see Materials and methods). Egg-laying animals were counted, the means were calculated, and the values are shown with SD. Upon recovery, *daf-2* PD adults were fertile, but *daf-2; aak(0)* PD adults were almost entirely sterile; ***P < 0.0001 using Marascuilo procedure. Assays were performed 3 times, and the data represent the mean ± SD; *n* = 50. (B) In *daf-2; aak(0)* PD animals, the highly penetrant sterility is also associated with vulval defects. A portion of these animals (16.5% ± 3.5%) prematurely expired during their recovery phase and failed to reach adulthood. Values represent means ± SD; *n* = 50. Underlying data can be found in S1 Data. *aak*, AMP-activated Protein Kinase subunit; DAF, DAuer Formation abnormal; PD, post-dauer; WT, wild type.

The dauer larva is remarkable in that it can remain in a quiescent state for months, while animals that develop through the reproductive mode (nondauer) die after 2–3 wk. Nevertheless, the dauer larva can exit this quiescence upon improvement in growth conditions to resume reproductive development with little to no compromise of their reproductive fitness regardless of the duration of the developmental arrest per se [27]. The germ cells must therefore retain the appropriate information to maintain their totipotency over lengthy periods so that upon recovery from the diapause, the animal can still reproduce without any loss in fitness. Since AMPK and LKB1 are critical to block germ cell divisions during the dauer stage, we questioned whether the supernumerary germ cells that are produced in *aak(0)* mutants are indeed competent to generate functional gametes and/or embryos. Following an SDS-based dauer selection [15], we quantified the brood size of *daf-2* (control) and *daf-2; aak(0)* animals after allowing both of these mutants to recover after having spent 24 h in the dauer stage. In contrast to *daf-2* animals that recover from dauer with very little to no negative reproductive consequence, AMPK mutant animals that transit through the dauer stage for 24 h or more exhibit highly penetrant post-dauer (PD) sterility upon recovery (Fig 1A and S2 Fig). The brood size of *daf-2* PD adults was not significantly different from *daf-2* animals that never transit through dauer (S2 Fig), suggesting that passage through the dauer stage has little to no impact on reproductive fitness provided that AMPK signaling is active. Moreover, both catalytic subunits are equally sufficient to ensure PD fertility since the fertility of *daf-2* mutants that lack either *aak-1* or *aak-2* is not significantly different from that of *daf-2* PD adults (S1A Fig).

Under normal growth conditions, *aak(0)* animals exhibit normal somatic and germline development and a high percentage of fertile animals (Fig 1A) but do have a slightly reduced brood size when compared to *daf-2* mutants (S2 Fig). The PD sterility of these mutants is therefore linked to their inability to adjust to the energy stress of the dauer stage. To determine whether the PD sterility we observed in the AMPK mutants is exclusive to the insulin-like signaling branch involved in dauer formation or whether it also occurs downstream of other dauer signaling pathways, we evaluated the fertility of *daf-7* mutants (Transforming Growth Factor-β [TGF-β] pathway) that lack AMPK signaling. We also assessed PD sterility in *aak(0)* mutants treated with dauer pheromone [25]. Similar to what occurs in the insulin-like signaling mutants, inducing the dauer stage through the compromise of the TGF-β pathway or treatment with dauer pheromone also results in highly penetrant sterility in PD animals that lack AMPK (S1 Fig). Thus, the activity of AMPK is critical for PD fertility, and hence the maintenance of germ cell integrity, downstream of the major pathways required for dauer formation.

Although most of the *aak(0)* PD animals become sterile, a significant portion die during recovery. Those that survive also show diverse abnormalities in vulva development (Fig 1B and S1 Table). Thus, it is not only the germ line that is compromised in PD animals that lack AMPK, but at least some somatic tissues are also sensitive to loss of AMPK function.

### AMPK PD gonads are morphologically abnormal, and the germ cells fail to exit pachytene

**Fig 2:**
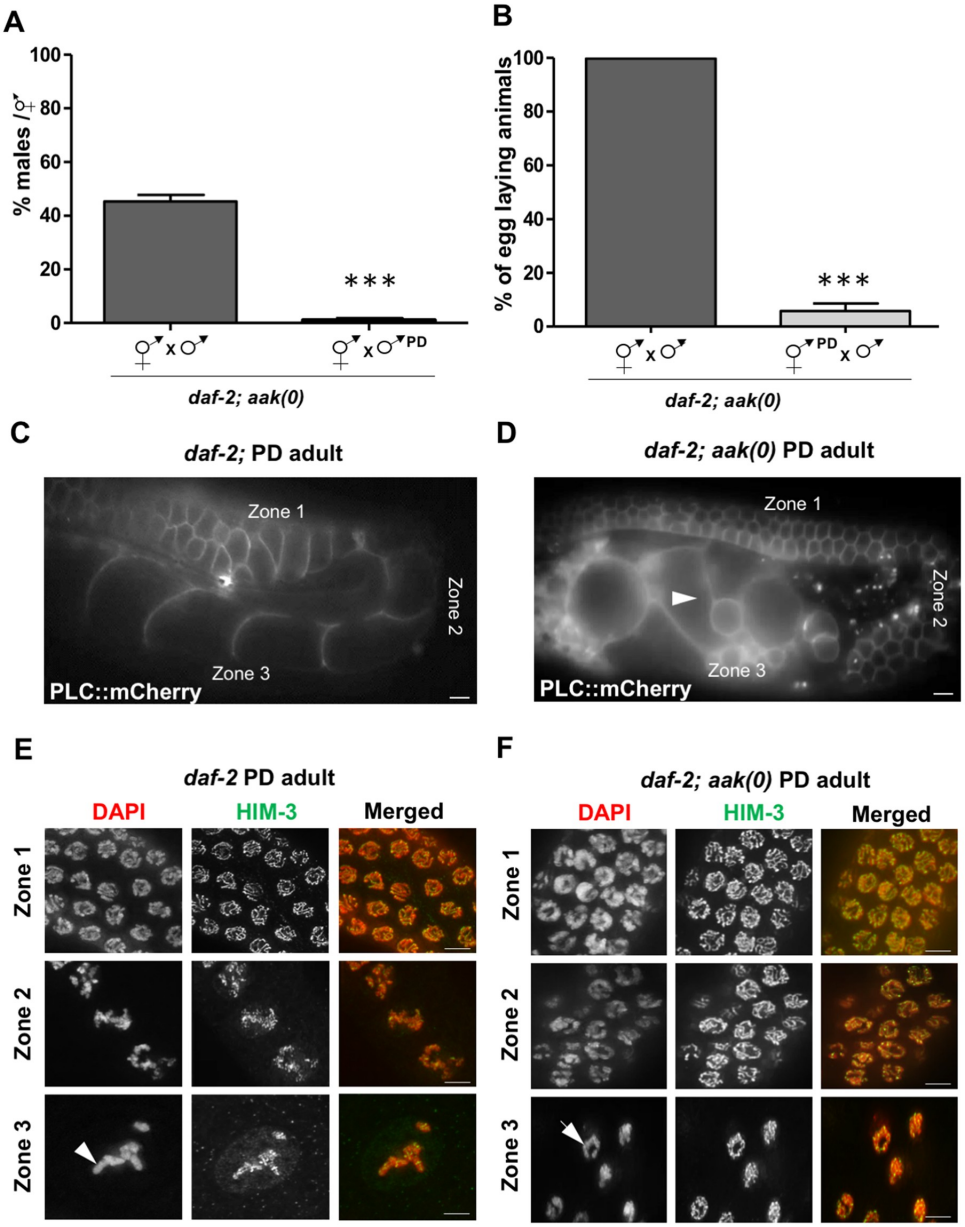
AMPK-defective PD adults show abnormal gonadal morphology, and the germ cells fail to exit pachytene. (A, B) Reciprocal crosses were performed, and a ratio of 20 males per hermaphrodite was maintained for all crosses. *daf-2; aak(0)* PD males were mated with control (never transited through the dauer stage) *daf-2; aak(0)*. 15 individual crosses were set up, and a percentage of males/hermaphrodite in the F_1_ were counted. Few to no male progeny were identified in the F_1_ generation. The mean is shown ± SD. (B) *daf-2; aak(0)* PD hermaphrodites were crossed with control (never transited through the dauer stage) *daf-2; aak(0)* males. 15 crosses were set up, and PD *aak(0)* hermaphrodites exhibited a high frequency of sterility. The mean is represented ± SD. ***P < 0.0001 using Marascuilo procedure. (C, D) All animals analyzed express a *Ppie-1*::PLC::mCherry transgene to monitor germ cell membranes/organization. (C) In *daf-2* PD adults, the gonad and germ cells developed normally and no obvious defects were observed, but *daf-2; aak(0)* PD adults exhibited various defects in gonad development and organization (D). Oocyte morphology was abnormal (white arrowhead), and they lacked the typical file-like organization observed in the control *daf-2* PD animals. (E, F) Meiotic progression was monitored by subdividing the post-transition zone gonad into 3 different subregions. In the first subregion after the transition zone (Zone 1), germ cells entered pachytene stage; in Zone 2, the cells did exit pachytene and initiated the separation of the paired chromosomes (diplotene); in Zone 3, separation of the paired chromosomes was complete, with 6 tightly condensed DAPI-stained bodies representing 6 pairs of homologous chromosomes (diakinesis). (E) In *daf*-2 PD, the germ cells passed through all these meiotic stages to eventually give rise to 6 condensed DAPI-stained bodies (white arrowhead). (F) In *daf-2; aak(0)* PD adults, the germ cells entered pachytene in Zone 1 but failed to completely exit the pachytene stage based on the continued presence of long chromosome tracks (white arrow). (C–F) *n* = 20. Scale bar: 10 μm in C and D, 4 μm in E and F. Underlying data can be found in S1 Data. *aak*, AMP-activated Protein Kinase subunit; AMPK, AMP Kinase; DAF, DAuer Formation abnormal; HIM-3, High Incidence of Males 3; PD, post-dauer; PLC, PhosphoLipase C; *Ppie*, Promoter of Pharynx and Intestine Excess.

To determine whether the sterility was a result of abnormal sperm formation or function or whether the defect was associated with the oocytes, we performed reciprocal crosses and monitored the brood size of the resulting cross progeny (Fig 2A and 2B). Using an antibody that detects sperm-specific proteins (anti-Major Sperm Protein [MSP]), we noted that while sperm was present in the *aak(0)* PD hermaphrodites, it may have been produced during the dauer stage [17]. To determine whether the sperm present in the *aak(0)* PD adults is functional, we mated *aak(0)* hermaphrodites that never transited through dauer with *aak(0)* PD males (a ratio of 20 males per hermaphrodite was maintained). If the mating were successful and the PD males produced functional sperm, we would expect approximately 50% of the progeny to be male. However, no significant F_1_ male progeny were observed, suggesting that the PD *aak(0)* sperm is defective (Fig 2A). We cannot rule out, however, that despite our monitoring, these mutants could be mating-incompetent.

Similarly, when we mated PD *aak(0)* hermaphrodites and *aak(0)* males that never transited through dauer, *aak(0)* PD hermaphrodites still exhibited highly penetrant sterility (Fig 2B), suggesting that integrity of the oocytes is also compromised. These results collectively suggest that germline development is sensitive to the energetic stress typical of dauer, and, in the absence of AMPK, the germ line becomes severely perturbed; the integrity of both the oocytes and the sperm is affected, ultimately rendering the PD animals sterile.

To further determine the physiological basis of the observed sterility in the *aak(0)* PD animals, we examined their gonad morphology and organization using a germ cell membrane marker [28]. We noted that in 95% of the animals, the oocyte morphology appeared abnormal, and they also lacked the typical single-file organization seen in the control *daf-2* PD animals (Fig 2C and 2D and S2 Table). Also, in 60% of the *aak(0)* PD animals, the general gonad symmetry was abnormal in terms of size and shape of the gonadal arms. (S2 Table).

We stained the germ lines of PD control and AMPK mutant animals and examined chromosome morphology within the germ cells as they progressed through the distinct regions of meiotic prophase to generate fully differentiated oocytes [29]. There were no obvious defects in the size of the mitotic zone or the spatiotemporal arrangement of the transition zone. For further characterization, we binned the post-transition zone germ cells into 3 different zones: in Zone 1, germ cells enter the pachytene stage; in Zone 2, the cells exit pachytene and initiate the separation of the paired homologous chromosomes (diplotene); in Zone 3, separation of homologues is complete, forming 6 tightly condensed DAPI-stained bodies representing 6 pairs of homologous chromosomes (diakinesis).

In the *daf-2* PD germ line, there were no observed abnormalities as the germ cells completed meiotic progression to eventually give rise to oocytes with 6 condensed DAPI-stained bodies (Fig 2E). However, in the PD germ lines of *aak(0)* mutants, the germ cells do enter pachytene in Zone 1 but then fail to exit Zone 2. The pachytene arrest persists into Zone 3 as the chromosomes fail to separate and condense (Fig 2F). This suggests that in *aak(0)* PD animals, the germ cells fail to exit pachytene and thus never undergo diakinesis to produce mature oocytes.

### Reducing germline hyperplasia does not restore fertility in PD AMPK mutants

**Fig 3:**
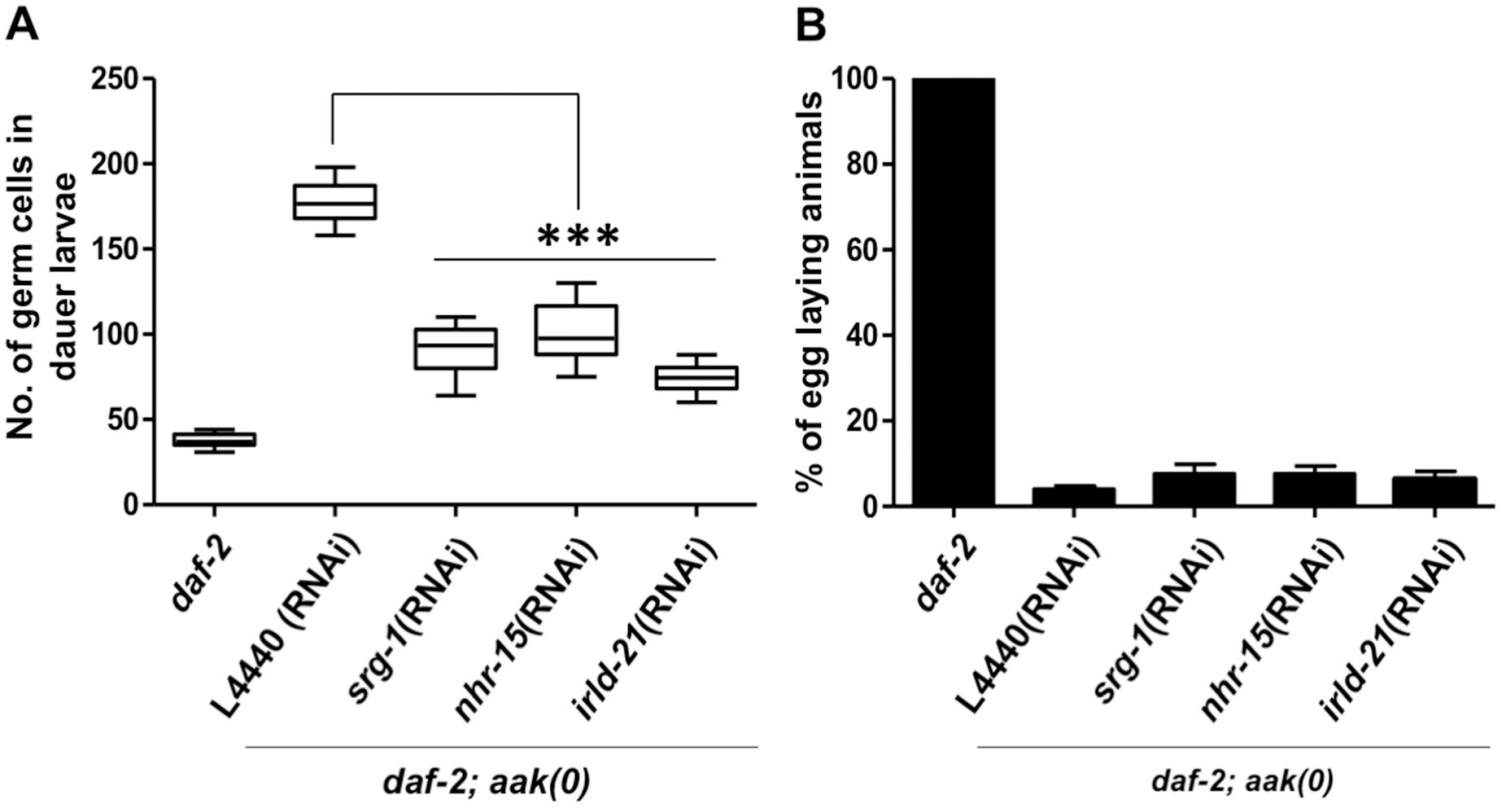
The PD sterility of AMPK mutants is not a direct consequence of germ cell divisions during the dauer stage. (A, B) Whole-animal DAPI staining was performed to quantify the number of dauer germ cells. The number of egg-laying animals was quantified, and the mean is represented ± SD. To test if the germ cell integrity defect results from the dauer-dependent germline hyperplasia, we used RNAi to disrupt 3 gene products previously found to suppress germline hyperplasia in dauer larvae [30]. Larvae were switched to permissive temperature to exit dauer and resume reproductive development. Fertility was assessed 48 h after the temperature shift by counting egg-laying adults. L4440 is an empty RNAi vector and is used as a control. ***P < 0.0001 when compared with L4440 using the two-tailed *t* test. *n* = 50. Underlying data can be found in S1 Data. *aak*, AMP-activated Protein Kinase subunit; AMPK, AMP-activated Protein Kinase; DAF, DAuer Formation abnormal; *irld*, Insulin/EGF-Receptor L Domain protein; *nhr*, Nuclear Hormone Receptor; PD, post-dauer; RNAi, RNA interference; *srg*, Serpentine Receptor, class G.

Because *aak(0)* dauer larvae fail to establish the GSC arrest and exhibit sterility upon exiting the dauer stage, we questioned whether the observed sterility might result from the inappropriate germ cell divisions that occur as animals enter the dauer stage. To test this, we performed RNA interference (RNAi) on genes that were shown to suppress the germline hyperplasia observed in the *aak(0)* dauer larvae [30]. We subsequently allowed these animals to recover to form adults, after which we assessed their fertility and brood size. None of the suppressors of dauer germline hyperplasia that we tested were capable of restoring the PD fertility in the *aak(0)* mutants, although the germline hyperplasia typical of *aak(0)* mutant dauer larvae was visibly ameliorated (Fig 3A and 3B). However, RNAi of these genes does not completely correct the germline hyperplasia, leaving the possibility that the supernumerary germ cells could be responsible for the PD sterility. Nevertheless, our data suggest that the PD sterility of *aak(0)* animals is not necessarily a direct consequence of the dauer germline hyperplasia but may involve additional processes that could act in concert with, but possibly independent of, the regulation of cell cycle quiescence during the dauer stage.

### Many chromatin marks are misregulated both in the soma and in the germ line in *aak(0)* dauer larvae

*C*. *elegans* dauer larvae exhibit a significantly different gene expression profile when compared to animals that never transit through the dauer stage [12]. Furthermore, these changes in gene expression persist after the animals exit dauer and become reproductive adults. Thus, a molecular memory of the passage through dauer is recorded and has been shown to influence fertility in the PD animals [12]. The observed changes in gene expression are highly correlated with the changes that occur in the various chromatin marks detected in dauer and in PD larvae and that are distinct from controls that never transited through the dauer stage [12].

AMPK has been implicated in the regulation of gene expression through its ability to modify chromatin by directly phosphorylating histone H2B to activate stress-promoted transcription [31]. Furthermore, we recently showed that AMPK modulates the chromatin landscape to ensure that transcriptional activity is blocked in the primordial germ cells until animals have sufficient cellular energy levels [5]. Since AMPK may directly regulate histone modifying enzymes to bring about changes in gene expression, we wondered whether chromatin modification may be perturbed in the dauer germ cells, thereby affecting the adaptive gene expression program that would normally occur in dauer. This inability to appropriately adjust to the energy stress associated with dauer development might explain the loss of integrity in the PD germ cells.

**Fig 4:**
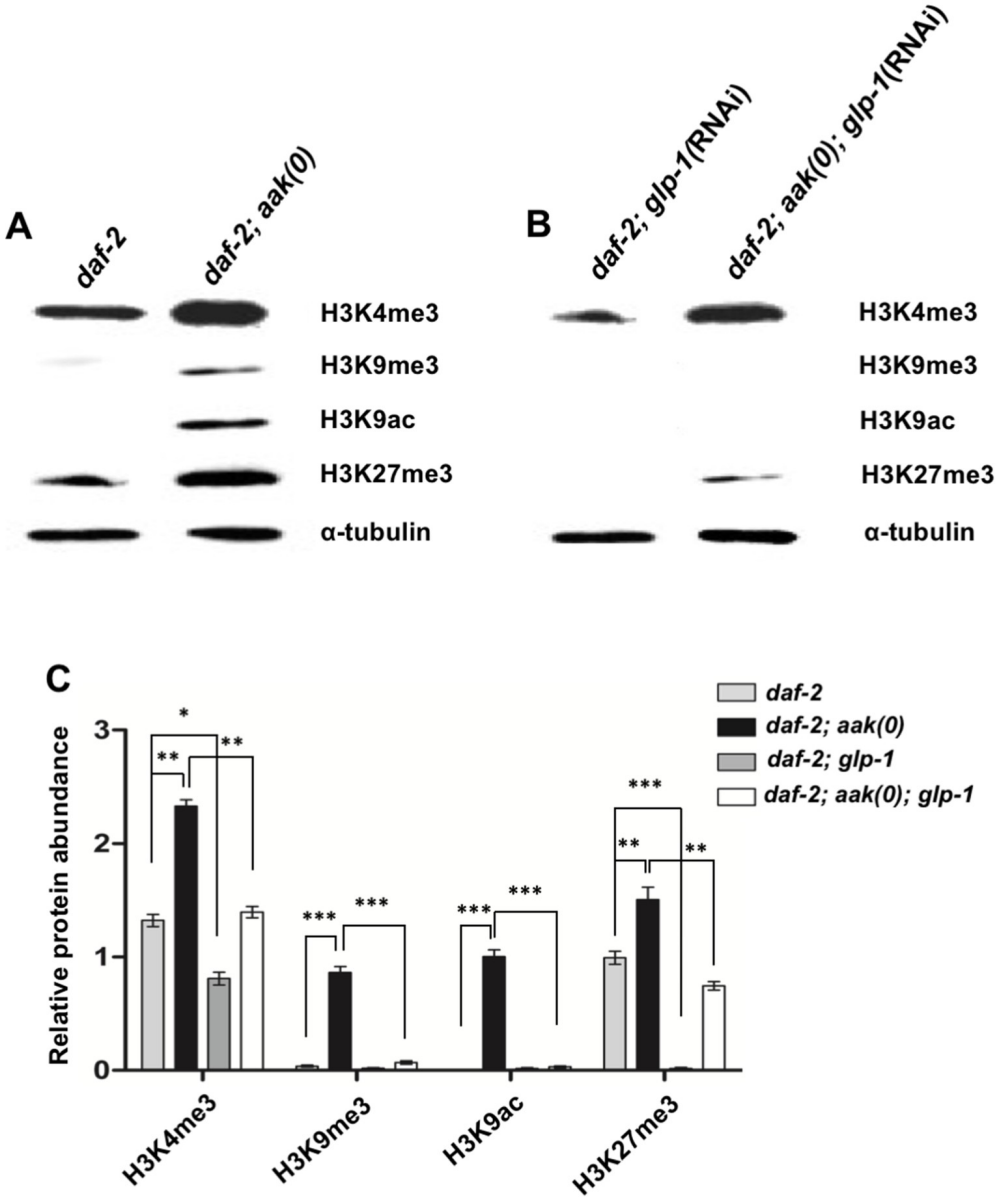
AMPK modulates the abundance of diverse chromatin marks in the soma and the germ line during the dauer stage. (A, B) Global levels of H3K4me3, H3K9me3, H3K9ac, and H3K27me3 were quantified by performing whole-animal western blot analysis of *daf-2* and *daf-2; aak(0)* dauer larvae. *glp-1*(RNAi) was performed postembryonically using dsRNA feeding in order to compromise germline development without affecting early embryogenesis. (C) Global levels of these chromatin marks were quantified using whole-animal western analysis. α-tubulin was used as a loading control to normalize protein levels between samples. Error bars indicate SD from 3 independent experiments. *P < 0.05, **P < 0.001, ***P < 0.0001 using Student’s *t* test. Underlying data can be found in S1 Data. *aak*, AMP-activated Protein Kinase subunit; AMPK, AMP Kinase; DAF, DAuer Formation abnormal; dsRNA, double-stranded RNA; *glp-1*, Germline Proliferation abnormal-1; H3K4me3, histone H3 lysine 4 trimethylation; H3K9ac, Histone H3 Lysine 9 Acetylation; H3K9me3, histone H3 lysine 9 trimethylation; H3K27me3, histone H3 lysine 27 trimethylation RNAi, RNA interference.

We therefore examined the global levels of diverse chromatin marks that were previously found to change following transit through the dauer program. We first performed western blot analysis on whole worm extracts from *daf-2* and *daf-2; aak(0)* mutant dauer larvae using antibodies specific for histone modifications that are associated both with transcription activation (histone H3 lysine 4 trimethylation [H3K4me3] and Histone H3 Lysine 9 Acetylation [H3K9ac]) and repression (histone H3 lysine 9 trimethylation [H3K9me3] and histone H3 lysine 27 trimethylation [H3K27me3]). Interestingly, all the marks we tested were abnormally high in the absence of AMPK (Fig 4A). To confirm whether the increased level of these chromatin marks was associated with the hyperplasia associated with AMPK mutant dauer larvae, we performed the same experiments in animals that lack GermLine Proliferation abnormal-1 (*glp-1*) (S3 Fig) [29] and quantified the levels of the chromatin marks. The reduction of germ cells significantly decreased the levels of the chromatin marks, suggesting that the germ cells are the major contributors to the global increase in the levels of the chromatin marks in AMPK mutant dauer larvae, although the levels also increase in the soma (Fig 4B).

### The distribution of chromatin marks is dramatically altered in *aak(0)* dauer germ cells and higher levels of these marks persist in the PD germ line

**Fig 5:**
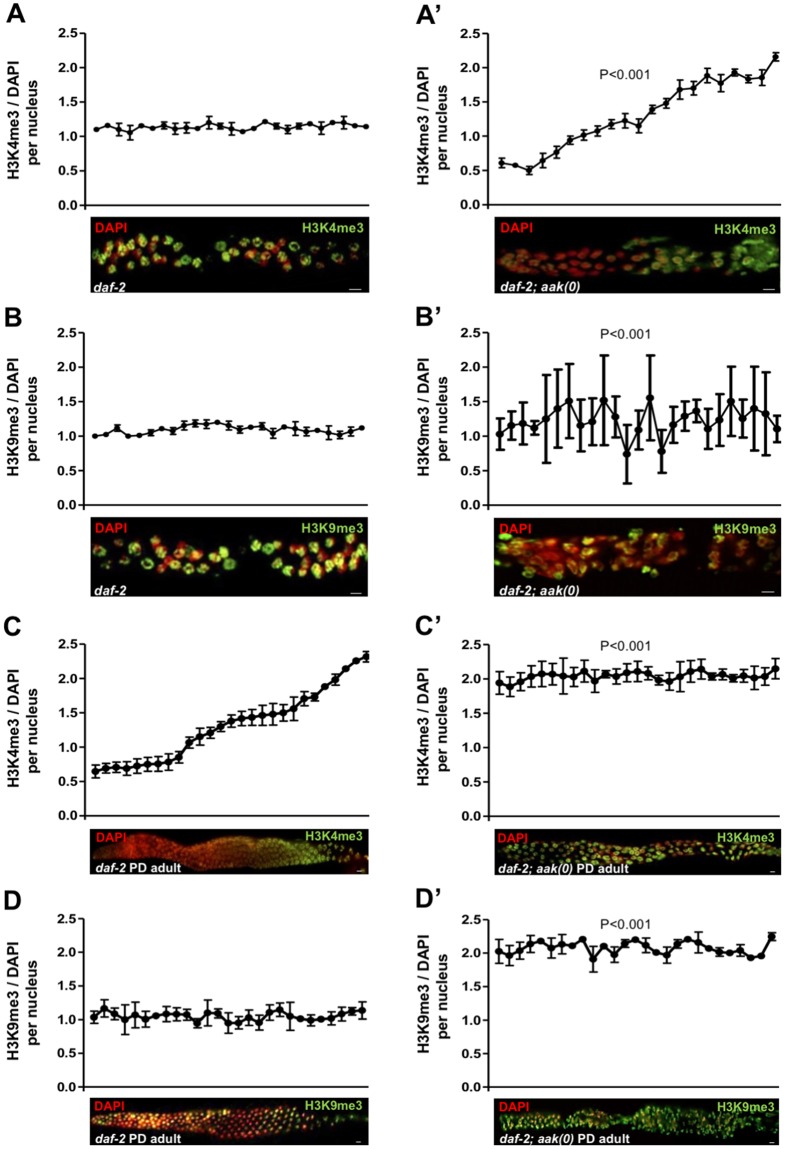
The distribution and abundance of both activating and repressive chromatin marks are dramatically altered in the *aak(0)* dauer and PD germ cells. All images are merged, condensed Z stacks. The graphs represent the average immunofluorescence signal of anti-H3K4me3 and anti-H3K9me3 normalized to DAPI across the dissected germ line. For the micrographs of *daf-2* dauer gonads, the entire dauer gonad was analyzed (distal, proximal, distal). Because of technical difficulties, only a single gonadal arm of the *daf-2; aak(0)* gonad was analyzed (distal, proximal). Images in A′, B′, C, C′, D, and D′ are aligned such that distal is left side and the proximal is right. (A, A′) The left panel (*daf-2*) and right panel (*daf-2; aak(0)*) show H3K4me3 (green), and in (B, B′), H3K9me3 (green) staining merged with DAPI (red). (C–C′, D–D′) PD *daf-2* and *daf-2; aak(0)* adult gonads were extruded and stained with anti-H3K4me3 and H3K9me3 (green), and signal intensity was quantified throughout the gonad using Image J software. **P < 0.001 using the F-test for variance when compared to *daf-2; aak(0)*. Scale bar: 4 μm, *n* = 15 for all the experiments. Underlying data can be found in S1 Data. *aak*, AMP-activated Protein Kinase subunit; DAF, DAuer Formation abnormal; H3K4me3, histone H3 lysine 4 trimethylation; H3K9me3, histone H3 lysine 9 trimethylation; PD, post-dauer.

From our western analysis, we could not discern whether the levels in the chromatin marks were abundant simply because of the supernumerary germ cells in the AMPK mutant dauer larvae or whether the levels were higher in each individual nucleus per se. We therefore dissected gonads from both *daf-2* and *daf-2; aak(0)* dauer larvae and quantified the levels of H3K4me3 and H3K9me3 to better evaluate their levels per nucleus and to determine whether there were any changes in their proximal–distal distribution through the gonad. In the control *daf-2* dauer germ line, the levels of both H3K4me3 and H3K9me3 are uniform across all nuclei throughout the dauer germ line, but in the AMPK mutant dauer larvae, the pattern of H3K4me3 and H3K9me3 expression in the gonad is altered, while their levels are highly variable in individual nuclei (Fig 5A and 5B). Of particular interest, we noted that the expression of H3K4me3 in individual nuclei is comparatively weak in the distal gonad but gradually increases in the proximal gonad, where it becomes much higher.

To test whether the abnormal distribution and abundance of the H3K4me3 and H3K9me3 marks are resolved after the *aak(0)* larvae exit dauer, we examined these marks in PD adult gonads. Interestingly, we noted that higher levels of both H3K4me3 and H3K9me3 persist in the *aak(0)* PD adult germ line when compared to the control *daf-2* PD animals (Fig 5C and 5D).

Altogether, these data confirm the role of AMPK in the regulation of both transcriptionally activating and repressive chromatin marks in the germ line under energetic stress. In its absence, the levels of each mark we tested increased while the distribution of these marks was dramatically altered.

### Gene expression is altered in both the *aak(0)* dauer and PD germ line

**Fig 6:**
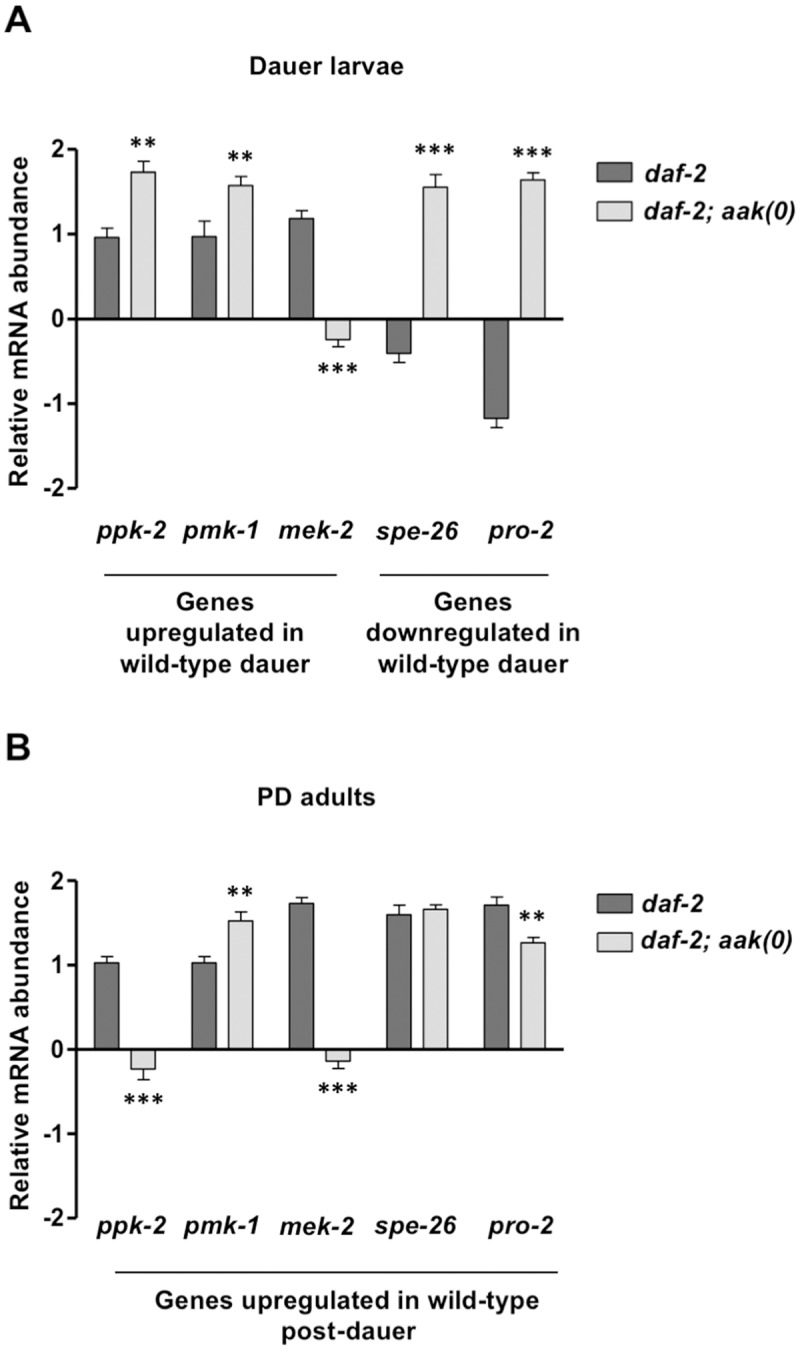
Gene expression is altered in both the *aak(0)* dauer and PD germ line. (A, B) Germline genes that were previously shown to be differentially expressed during and after transit through the dauer stage [12] were quantified in *daf-2* and *daf-2; aak(0)* dauer and PD animals. The relative mRNA levels were analyzed using quantitative real-time PCR in both *daf-2* and *daf-2; aak(0)* dauer and PD adults. The expression of these germline genes was significantly altered in *daf-2; aak(0)* dauer and PD animals when compared to *daf-2*. *tba-1* was used to normalize levels between samples. Error bars indicate SD from 3 independent experiments. **P < 0.001 using one-way ANOVA when compared to *daf-2*. Underlying data can be found in S1 Data. *aak*, AMP-activated Protein Kinase subunit; DAF, DAuer Formation; *mek*, MAP kinase kinase or Erk Kinase; PD, post-dauer; *pmk*, P38 Map Kinase family; *ppk*, PIP kinase; *pro*, proximal proliferation in germ line; *spe*, SPErmatogenesis; *tba*, TuBulin Alpha.

To examine whether aberrant chromatin modifications result in abnormal gene expression in animals that lack AMPK signaling, we performed quantitative Reverse Transcription Polymerase Chain Reaction (qRT-PCR) to quantify the transcript levels of selected germline-expressed genes that were found to be significantly altered in dauer and in the PD adults compared to animals that never transited through dauer [12]. The abundance of these transcripts was considerably different in *aak(0)* dauer larvae; some of the genes (*ppk-2*, *pmk-1*, *spe-26*, *pro-2*) were present at higher levels, while *mek-2* was detected at significantly lower level when compared to control *daf-2* animals (Fig 6A). Furthermore, some of these differences in transcript abundances are not resolved in the PD adult germ line of AMPK mutants (Fig 6B). Therefore, the chromatin modifications that we observed in both the dauer and PD animals that lack all AMPK signaling may prompt a dramatic deviation in the gene expression program that would normally occur in the germ line as a result of transit through the dauer stage.

### Compromise of endogenous siRNA pathway components partially suppresses *aak(0)* PD sterility and dauer germline hyperplasia

Many endogenous small RNAs are critical in distinguishing loci to be targeted by chromatin modifying enzymes, in addition to specifying whether the modification will be active or repressive. They act as mediators of gene expression in order to adapt to cell-type information and to varying environmental situations [32]. It is therefore not surprising that the small RNA repertoire is dramatically altered in both dauer and PD adults compared to animals that develop in a replete environment [13]. We were therefore curious to know whether AMPK might regulate the changes that occur in the suite of small RNA species during dauer, considering its role in regulating chromatin marks in response to energetic stress. To test this possibility, we used RNAi to compromise critical components of the microRNA (miRNA) (Alg1 INteracting protein [*ain*]-*1*), the germline/nuclear RNAi (Heritable RNAi Deficient [*hrde*]*-1*, Chromosome-Segregation and RNAi deficient [*csr*]*-1*) pathways, and some common upstream effectors that impinge on all the small RNA pathways (DiCer Related [*dcr*]*-1*, RNAi DEfective [*rde*]*-4*) to determine whether disabling any of these pathways might affect the sterility of the PD AMPK mutant adults.

**Fig 7:**
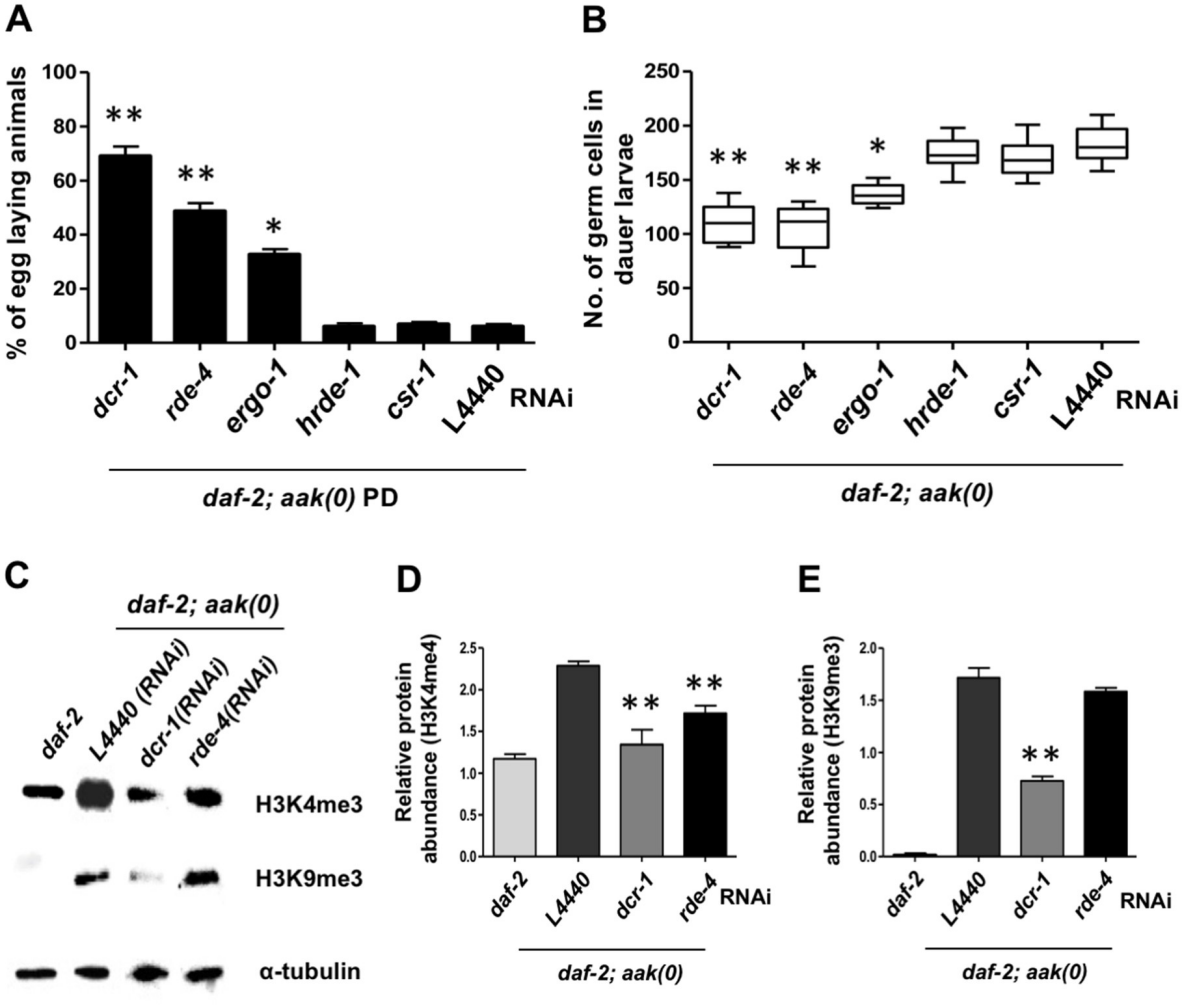
Compromise of small RNA pathway components partially suppresses *aak(0)* PD sterility and dauer germline hyperplasia. To compromise the function of the small RNAi pathway, *daf-2; aak(0)* animals were subjected to RNAi by dsRNA feeding against multiple components of the endogenous RNAi pathway. The L4440 empty RNAi vector was used as a control. (A) The PD sterility observed in the *daf-2; aak(0)* animals was partially rescued by *dcr-1*, *rde-4*, and *ergo-1* RNAi, while RNAi for the germline nuclear Argonautes *csr-1* and *hrde-1* failed to suppress the observed sterility. **P < 0.001 and *P < 0.05 using Marascuilo procedure, and *n* = 100. (B) Whole-animal DAPI staining was performed to quantify the number of germ cells and the germline hyperplasia in the *daf-2; aak(0)* dauer larvae. Statistical analysis was performed using the two-tailed *t* test when compared to L4440-treated animals, where **P < 0.001 and *P < 0.05; *n* = 100. (C, D, E) Following the RNAi treatment, global levels of H3K4me3 and H3K9me3 were quantified using whole-animal western analysis. α-tubulin was used as a loading control to normalize protein levels between samples. Error bars indicate SD from 3 independent experiments. **P < 0.001 using Student’s *t* test. Underlying data can be found in S1 Data. *aak*, AMP-activated Protein Kinase subunit; *csr-1*, Chromosome-Segregation and RNAi deficient 1; DAF, DAuer Formation abnormal; *dcr-1*, DiCer Related 1; dsRNA, double-stranded RNA; ERGO-1, Endogenous-RNAi–deficient arGOnaute 1; *hrde-1*, Heritable RNAi Deficient 1; H3K4me3, histone H3 lysine 4 trimethylation; H3K9me3, histone H3 lysine 9 trimethylation; PD, post-dauer; *rde-4*, RNAi DEfective 4; RNAi, RNA interference.

AMPK mutants were therefore grown on IPTG (RNAi-inducing) plates until they all entered the dauer stage. After 24 h, they were allowed to resume normal development on regular Nematode Growth Medium (NGM; non-RNAi–inducing) plates to study their PD fertility. The miRNA pathway is essential for dauer entry, making it difficult to interpret their potential role in this process [33]. On the other hand, we found that the individual disruption of the RNase III-like *dcr-1*, its accessory factor RDE-4, or the primary Argonaute protein Endogenous-RNAi–deficient arGOnaute (ERGO-1) could partially rescue the sterility of PD AMPK mutants and the uncontrolled proliferation in the germ line of AMPK mutant dauer larvae, in addition to some of the somatic defects (Fig 7A and 7B, S2 Fig, and S1 Table). In contrast, the compromise of the nuclear Argonaute proteins HRDE-1 or the germline licensing Argonaute CSR-1 had little effect on the hyperplasia or the PD sterility of the AMPK mutants. These data indicate that AMPK must directly or indirectly regulate an endogenous small RNA pathway that affects both GSC proliferation and integrity but may not include the canonical nuclear Argonaute proteins that have been characterized to regulate germline gene expression. We cannot, however, rule out that the RNAi effect on either of these two nuclear Argonautes is efficient enough to elicit a phenotypic effect [34].

To validate our RNAi results, we crossed mutations in *dcr-1*, *rde-4*, *ergo-1*, and *hrde-1* into the AMPK mutant background and determined the PD sterility in each of the compound mutants. *csr-1* has recently been shown to be required for dauer formation [35] and therefore could not be further characterized in our study. With the exception of *dcr-1*, neither the RNAi nor the mutations of these small RNA pathway components affected development or fertility when combined with AMPK mutants under normal growth conditions (S4C and S4D Fig). However, unlike the RNAi treatments that partially restored PD fertility, none of these alleles suppressed the sterility typical of the AMPK mutant PD animals. Although this observation is curious, it is not uncommon and has been observed in other contexts that involve epigenetic modification [36]. This apparent inconsistency most likely reveals a more complex role of these small RNA components, perhaps in more than one aspect of dauer development.

Our RNAi protocol addressed the role of these gene products during the dauer stage, but they may be equally important during PD recovery. But, because dauer recovery is performed on non-RNAi plates, the small RNA components involved in recovery may attain a threshold level of function capable of supporting the chromatin remodeling necessary for PD fertility. This threshold activity would be entirely absent in the genetic mutants and might account for the differences between the RNAi phenotypes and the genetic mutants.

If this were true, then continuously subjecting the animals to RNAi during the recovery period while also enhancing the RNAi effect by increasing the IPTG concentrations might phenocopy the observations we obtained with the genetic mutants. This was indeed the case; under these recovery conditions, none of the small RNA pathway components suppressed the sterility of the AMPK PD animals and behaved very similar to their corresponding genetic mutations (S4 Fig). These data suggest that attenuating the small RNA–pathway function during entry while allowing a specific threshold level of activity during recovery is sufficient for the partial reestablishment of fertility in the PD AMPK mutants.

Since both the dauer-associated hyperplasia and the PD sterility of the AMPK mutants correlated with the misregulation of chromatin modifications, we wanted to confirm whether the compromise of these various RNAi pathway components might also restore the inappropriate levels and distribution of the chromatin marks in the AMPK mutant germ line. We therefore quantified the global levels of both H3K4me3 and H3K9me3 in *dcr-1*(RNAi)–and *rde-4*(RNAi)–treated dauer larvae. The levels of both of these marks were significantly reduced in the *dcr-1*(RNAi) AMPK mutant dauer animals. Surprisingly, only the level of H3K4me3 was significantly reduced in the *rde-4*(RNAi) AMPK mutants (Fig 7C, 7D and 7E), although this may reflect the weak and variable RNAi penetrance typical of *rde-4*. These data confirm that AMPK impinges on the endogenous small RNA pathway to modulate chromatin modifications that affect both GSC proliferation and integrity, although it is currently unclear how AMPK might control these processes and whether phosphoregulation of key targets might be involved.

### Somatic AMPK activity is sufficient to regulate dauer germ cell quiescence and integrity through the transmission of small RNAs

**Fig 8:**
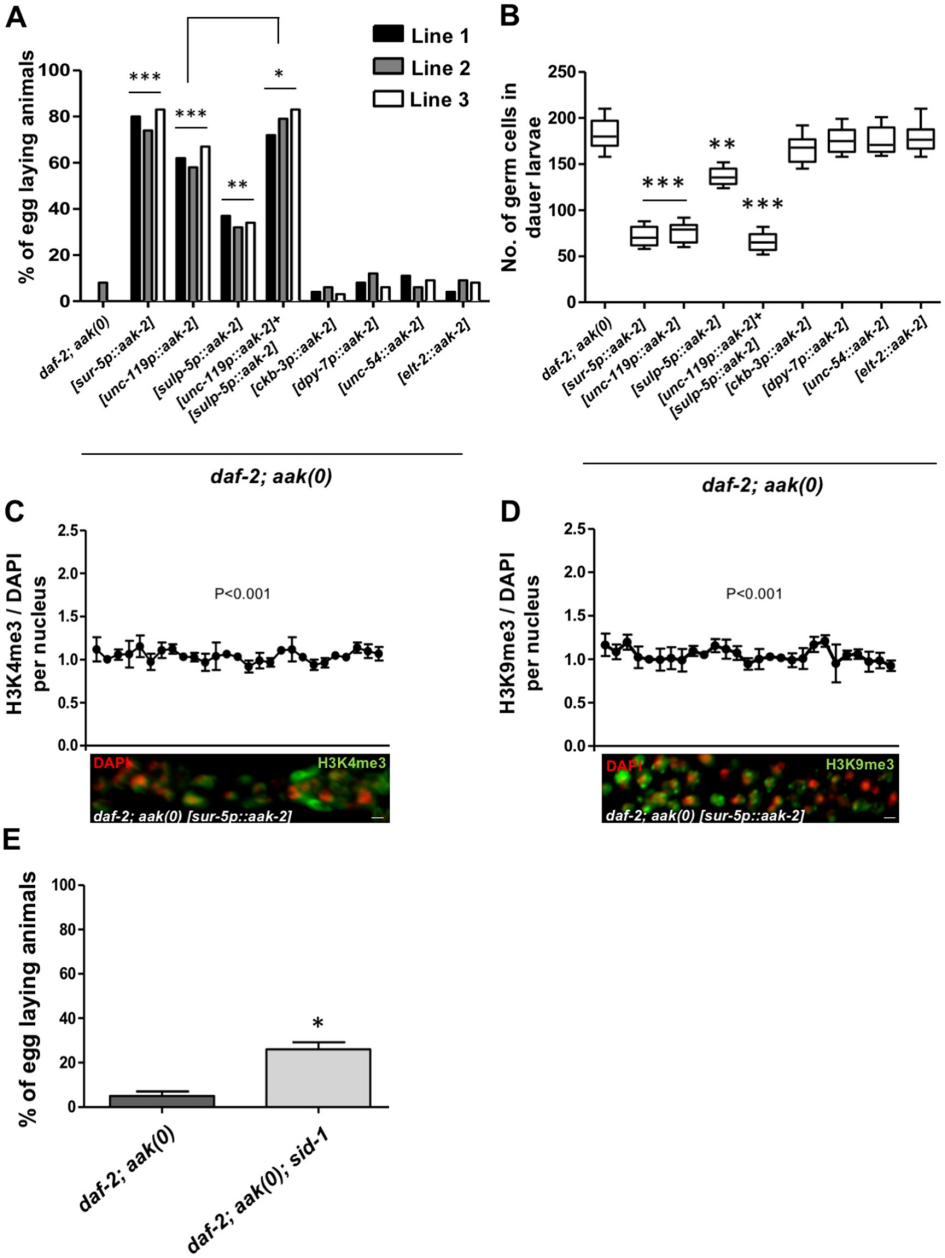
Somatic AMPK activity is sufficient to restore germ cell quiescence and integrity through the transmission of small RNAs in *aak(0)* mutants. (A) Plasmid constructs that contain *aak-2* cDNA driven by tissue-specific promoters were injected into *daf-2;aak(0)* mutants, and both the dauer-dependent germline hyperplasia and the PD sterility were evaluated for each transgenic strain. All transgenic lines are extrachromosomal and are represented by square brackets, and 3 independently generated lines were used for quantification. PD fertility was assessed 24 h following the temperature shift after animals were maintained minimally 24 h in dauer. *P < 0.05 when compared to [*unc-119p*::*aak-2*]; ***P < 0.0001 and **P < 0.001 using Marascuilo procedure when compared to *daf-2; aak(0)*; *n* = 50. (B) Whole-animal DAPI staining was performed to quantify the number of germ cells present in the dauer gonad in the transgenic lines and compared to controls. ***P < 0.0001 and **P < 0.001 using the two-tailed *t* test when compared to *daf-2; aak(0)*; *n* = 50. (C, D) All the analyzed images are merged, condensed Z stacks. The graphs represent the average immunofluorescence for H3K4me3 and H3K9me3 normalized to DAPI across the dissected gonad. **P < 0.001 using F-test of variance when compared to *daf-2; aak(0)*, and *n* = 10. (E) Disrupting soma-to-germline transmission of dsRNA by compromising the function of *sid-1* partially restores fertility in the *daf-2; aak(0)* PD animals. A number of animals laying eggs were counted, and the mean is shown ± SD. *P < 0.05 using Marascuilo procedure when compared to *daf-2; aak(0)*, and *n* = 100. Underlying data can be found in S1 Data. *aak*, AMP-activated Protein Kinase subunit; AMPK, AMP Kinase; *ckb*, Choline Kinase B; DAF, DAuer Formation abnormal; *dpy*, DumPY; dsRNA, double-stranded RNA; Erythroid-Like Transcription factor family; H3K4me3, histone H3 lysine 4 trimethylation; H3K9me3, histone H3 lysine 9 trimethylation; No., number; PD, post-dauer; RNAi, RNA interference; *sid-1*, Systemic RNAi Defective; *sulp*, SULfate Permease family; *sur-5*, SUppressor of activated *let-60* Ras; *unc*, UNCoordinated.

Recent studies have shown that AMPK can act cell nonautonomously to regulate life span, and the L1 survival of AMPK mutants is greatly improved when AMPK is restored in neurons [37, 38]. To determine whether AMPK plays a nonautonomous role in maintaining GSC quiescence and integrity, we expressed the catalytic subunit of AMPK (*aak-2*) ubiquitously in the soma (SUppressor of Ras 5 Promoter [*sur-5p*]) of *aak(0)* mutants. Interestingly, ubiquitous somatic expression of *aak-2* restored the fertility in the PD *aak(0)* mutants and also rescued the dauer germline hyperplasia (Fig 8A and 8B and S2 Fig). This suggests that AMPK activity in the soma is sufficient to maintain the integrity and quiescence in the germ cells during the dauer stage. To determine in which somatic tissue AMPK function can restore quiescence and integrity to the GSCs during the dauer stage, we used tissue-specific promoters to express the major AMPK catalytic subunit (*aak-2*) and quantified PD sterility and the degree of germline hyperplasia in these transgenic strains. We therefore generated AMPK-null mutants that express *aak-2* exclusively in the neurons (UNCoordinated 119 [*unc*]-*119*), the excretory system (SULfate Permease family [*sulp*]-*5*), the somatic gonad (Choline Kinase B [*ckb*]-*3*), the hypodermis (DumPY 7 [*dpy*]-*7*), the gut (Erythroid-Like Transcription factor family [*elt*]-2), and the muscles (*unc-54*) [39, 40]. Surprisingly, only the restoration of AMPK function in the neurons and the excretory system partially rescued the fertility and GSC quiescence (Fig 8A and 8B). Expression of AAK-2 simultaneously in the neurons and the excretory system significantly increased the percentage of the fertile animals compared to the individual tissue-specific expression strains (Fig 8A). These observations suggest that AMPK expression in either or both of these two tissues is sufficient to control germ cell homeostasis cell nonautonomously during the energetic stress typical of the dauer stage.

Ubiquitous somatic expression of *aak-2* also restored the normal levels and distribution of H3K4me3 and H3K9me3 marks in the dauer germ line (Fig 8C and 8D, compare with Fig 5C′ and 5D′). These data confirm that the somatic function of AMPK can reestablish quiescence and maintain germ cell integrity through its effects on the germline chromatin landscape in dauer larvae that otherwise lack all AMPK signaling.

To further investigate whether AMPK modulates some aspect of soma-to-germline communication through the deployment of somatically derived small endogenous RNAs, we tested whether the double-stranded RNA (dsRNA) importer Systemic RNAi Defective (*sid*)*-1* could affect the PD sterility of AMPK mutants by impairing the uptake of small dsRNAs [41]. If the small RNA components that are regulated by AMPK are generated in the germ line to mediate the adaptive redistribution of the chromatin modifications and consequently the germline integrity of these animals, then the loss of a dsRNA importer like SID-1 should have no effect. However, the loss of *sid-1* partially restored fertility in AMPK PD animals (Fig 8E), suggesting that an AMPK-dependent control switch becomes misregulated in these mutants, allowing the transfer of an aberrant population of small endogenous RNAs to the germ line and culminating in the establishment of an inappropriate chromatin landscape. The resultant gene expression may therefore not reflect the necessary metabolic adjustment that must occur during the dauer stage. This would presumably alter the ability of the germ cells to adapt to the energy stress associated with dauer, ultimately compromising their integrity, resulting in PD sterility.

## Discussion

During the long periods of energetic stress associated with the dauer stage, AMPK becomes highly active, thereby regulating a plethora of cellular processes to enhance catabolism and maintain cellular/organismal homeostasis during the diapause. The loss of AMPK during this and other contexts of metabolic stress results in pleiotropic phenotypes that range from inappropriate behaviors to lethality [17, 39, 42]. The germ line is particularly sensitive to loss of AMPK, and the hyperplasia that we observe in AMPK mutants occurs as the animals prepare to enter the diapause [17]. The consequences of these unscheduled germ cell divisions have never been interrogated, but if the additional germ cells were competent, the excessive proliferation could potentially result in a significant increase in reproductive fitness. Alternatively, if the supernumerary cells were abnormal, it would be detrimental for subsequent generations.

We show that most of the germ cells that are produced in AMPK mutant animals are incompetent to generate functional gametes after even a transient passage through the dauer stage, dramatically diminishing the subsequent possibility of reproducing. AMPK signaling is therefore critical to coordinate germ cell quiescence and the appropriate metabolic adjustment required to maintain the developmental plasticity necessary to survive the often-lengthy organismal energy stress associated with dauer.

This role of AMPK may not be restricted to the germ cells. The vulval precursor cells must also undergo quiescence and maintain a state of developmental plasticity throughout the dauer stage, only to recommence their divisions and final steps of differentiation during dauer exit [43, 44]. The PD vulval defects we observe in the recovered AMPK mutant dauer larvae are consistent with this additional role of the kinase outside the germ line. It is quite plausible that AMPK cooperates with FOrkhead boX O (FOXO)/DAF-16 to maintain this state throughout the diapause and/or in other situations of energy stress.

Following dauer recovery, *daf-2* animals develop normally with little to no consequence on their reproductive fitness. However, the germ cells of the AMPK mutant PD animals become stalled in a pachytene-like state, very similar to mutants in the Mitogen Activated Protein Kinase (MAPK) pathway [29]. Although, morphologically, many of the proximal germ cells appear to undergo cellularization and oogenesis, at the nuclear level, they fail to complete diakinesis to form 6 condensed nuclear bodies, a hallmark of a mature oocyte [45]. Recently, AMPK was shown to regulate the MAPK pathway to block the germ cell divisions under nutritional stress, while compromise of Kinase Suppressor of Ras 1 (*ksr-1*), a negative regulator of Rat Sarcoma (Ras) signaling, also results in germline hyperplasia in an AMPK-dependent manner [30, 46–48]. Consistent with this, our gene expression analysis indicated that genes in the MAPK pathway were expressed abnormally, and this misregulation might indeed contribute to the *aak(0)* PD phenotype. Therefore, upon activation, AMPK could impinge upon key components of the MAPK pathway to modulate meiotic cell cycle progression, thereby delaying the production of mature gametes to ensure that reproductive development is coordinated with transit through the dauer stage.

In the absence of AMPK signaling, germ cell integrity is forfeited during even a brief passage through dauer, resulting in highly penetrant sterility in PD adult animals. But what might constitute germ cell integrity and how might it be maintained by AMPK over the duration of the dauer stage? Certainly, changes in gene expression play a significant role in mediating the adaptive cellular and metabolic adjustments necessary to endure the energetic stress of the dauer stage, whether it lasts 24 h or 24 d. As *C*. *elegans* larvae enter the dauer stage, the chromatin is remodeled, altering gene expression significantly [12, 49]. These modifications are tightly correlated with changes in the small RNA repertoire. Based on the accepted mechanism of small RNA–mediated effects on the chromatin, changes in the small RNA population likely presage chromatin remodeling, which together will constitute a molecular memory of this life history event [12]. The resulting marks generate a chromatin-based directive for the consequent establishment of distinct adaptive cellular responses or behavior(s).

The normal distribution of the H3K4me3 and H3K9me3 chromatin marks become visibly perturbed within the AMPK mutant dauer germ line. Moreover, the aberrant marks fail to resolve upon dauer exit, persisting into the adult PD germ line. Although we have not identified the penultimate AMPK target(s) that mediate these changes in chromatin regulation, the relationship between AMPK and chromatin regulators may be akin to its role during the L1 diapause, in which germ cell integrity is compromised because of irregular chromatin modifications that take place in the primordial germ cells in the absence of AMPK [5].

Curiously, both activating and repressive chromatin modifications become misregulated in the AMPK mutant dauer germ line. The enzymes responsible for these antagonistic histone modifications are dramatically different and do not share common protein components [50]. The target of AMPK may therefore be something other than the methyltransferases per se but rather a regulator of a common cofactor necessary for all these methyltransferases, such as S-adenosyl methionine [51]. Alternatively, the aberrant abundance and distribution of both H3K4me3 and H3K9me3, and perhaps other methylation marks, could also result from a defect in demethylation via enzymes like Lysine-Specific Demethylase 1 (LSD1)/Suppressor of PResenilin 5 (SPR-5) [52] that may require AMPK for activation during the dauer stage.

The observed anomalies in both the abundance and the distribution of the activating and repressive marks would perturb the coordination of germline gene expression with the energy stress of dauer. This would normally be mediated through a specific chromatin syntax in both dauer and PD AMPK animals. In the absence of AMPK, gene expression may no longer correspond to that of a germ cell or at least of a germ cell that has been subjected to the challenge of surviving severe energy stress. The inability of the gene expression program to adjust to the metabolic challenge is most probably responsible for the abnormal gonad and germline development, in addition to the somatic defects observed in the AMPK PD adults.

The germline abnormalities that we observe in AMPK mutants are dependent on the inappropriate regulation of components of a small RNA pathway. Although small RNA pathways have previously been linked to transcriptional and chromatin modification, our data indicate that these regulatory mechanisms are under direct or indirect control by AMPK during periods of energy stress.

Our fortuitous use of RNAi to evaluate the effects of these the small RNA–pathway components in regulating the chromatin remodeling required during dauer development allowed us to identify a compromised level of activity through which we could demonstrate an AMPK-dependent involvement in this small RNA–mediated process. This “sweet spot” that could only be achieved by RNAi analysis was also identified while examining the effects of a Jumonji-like histone demethylase, in which again, complete elimination of the remodeling enzyme had no phenotype [36]. In the context we describe, an adjustment in the small RNA repertoire is likely critical to establish a quiescent state to protect germ cells during a potentially lengthy diapause. This would require an AMPK-mediated modification in gene expression through the silencing and activation of specific genes downstream of context-dependent Argonaute protein function [35]. Because reducing the levels of the pathway components ameliorates the sterility, it is most probable that the levels of a subset or all small RNAs might become abnormally high in the AMPK mutant dauer germ line, accompanied by an inability to adapt to the pending energy challenge.

Upon reception of favorable growth cues, the resumption of reproductive development may require a second remodeling event that would again involve context-specific Argonaute proteins and their cognate small RNAs to establish a gene expression program typical of PD adults. This remodeling event may be independent of AMPK or at least coincident with its inactivation.

Although we have not yet identified the key AMPK targets that mediate this small RNA function, it is noteworthy that both Dicer and RDE-4 contain multiple consensus AMPK phosphorylation sites. Alternatively, in addition to the primary Argonaute protein ERGO-1, more of the uncharacterized Argonaute family members may be sensitive to AMPK signaling and contribute to this small RNA–mediated change in the chromatin landscape that occurs during dauer and PD recovery in *C*. *elegans*.

Using RNA-dependent RNA polymerase Family 1 (*rrf-1*) to address where AMPK is required, we initially concluded that AMPK acted in a germline-autonomous manner to maintain GSC quiescence in the dauer larvae [17]. The technical shortcomings of this strategy have since been well documented [50], and our recent transgenic experiments confirm that AMPK activity is sufficient in the neurons or the excretory system to regulate germ cell quiescence and integrity. The simultaneous restoration of *aak-2* in both the neurons and the excretory system significantly improved PD fertility compared to either of the individual partial-rescuing transgenes. It remains unclear whether or how these tissues communicate with one another and how they coordinate the downstream changes in the germ line.

**Fig 9:**
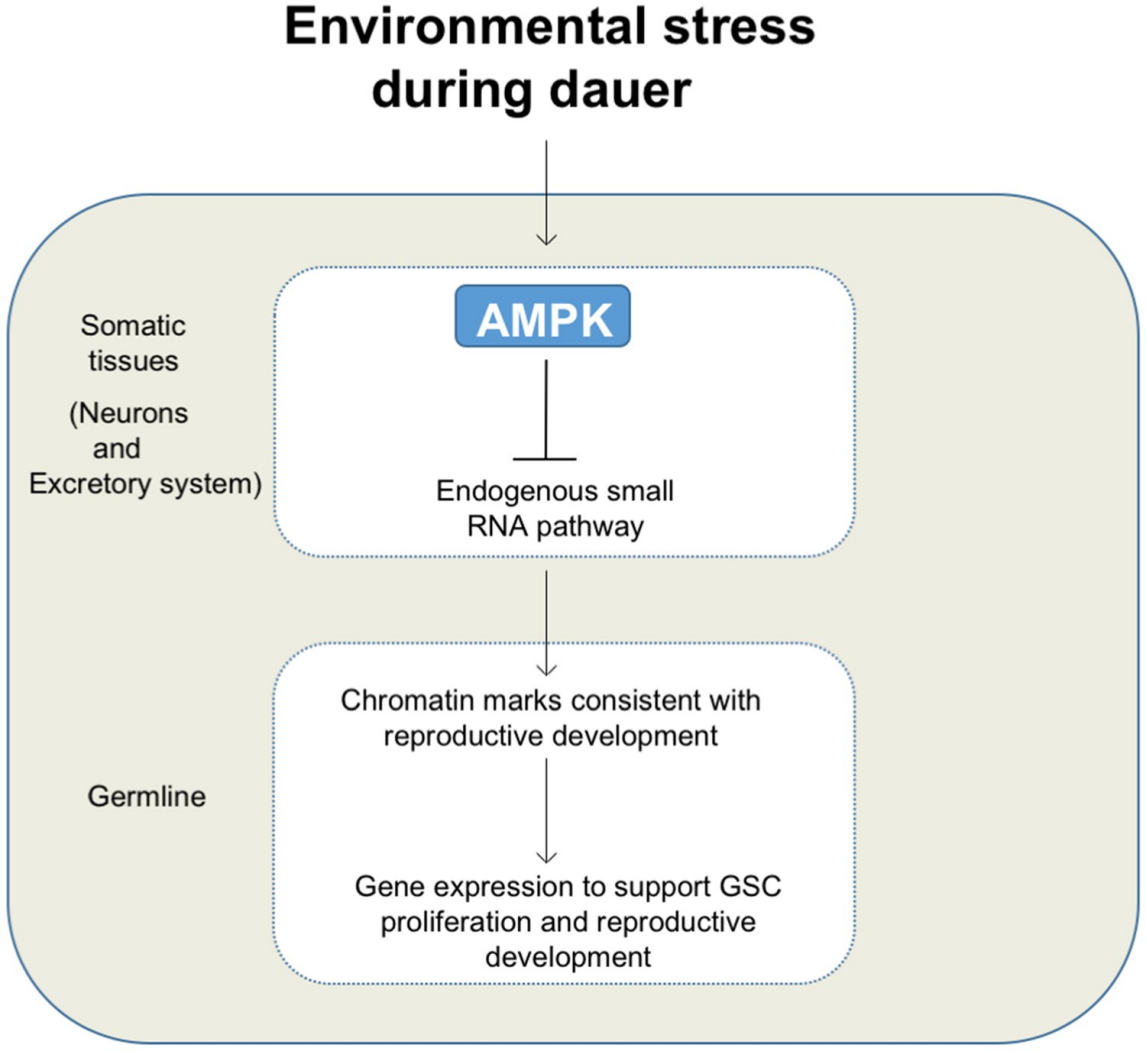
Regulation of GSC quiescence and integrity during dauer development. Upon encountering environmental stress, animals will enter the dauer stage. Consequent activation of AMPK in the somatic tissues can regulate a small RNA pathway to modulate dauer-specific chromatin modification in the dauer germ line. This maintains quiescence and integrity of the germ line and ensures reproductive fitness upon recovery from the diapause. Arrows and bars represent positive and negative interactions, respectively. AMPK, AMP-activated Protein Kinase; GSC, germline stem cell.

The ubiquitous expression of *aak-2* in the soma also restored the chromatin modifications to wild-type levels and a normal pattern of distribution throughout the dauer germ line. This may be entirely due to the combined effects of neuronal and excretory system expression. This neuron-specific function of AMPK may be a context-specific feature of AMPK signaling, since its neuronal expression failed to suppress the supernumerary germ cell divisions in AMPK mutants during the L1 diapause [35], but it was sufficient to extend life span in *C*. *elegans* under energetic stress [35, 36]. The neurons are the perfect sensors of the environment and can signal accordingly, perhaps in a neuroendocrine manner, to other tissues in order to adapt synchronously as an organism. AMPK could be one of the intermediates in transducing the signals from the neurons, potentially regulating some diffusible molecule to enhance survival without any compromise on their fitness. This would place AMPK at a critical position in sensing environmental challenges to ultimately impinge on the germ line to give rise to the observed chromatin-mediated adaptations associated with dauer and subsequent recovery in replete conditions (Fig 9).

But what neuron-derived diffusible signal could affect the chromatin in the germ cells? Like in many plants, RNAi is systemic in *C*. *elegans*. Injection of dsRNA into the somatic tissue can result in RNA-mediated gene silencing in the germ line [41, 53, 54]. Furthermore, the endogenous small interfering RNA (endo-siRNA) pathway that is active in the somatic tissues can contribute, at least in part, to the changes in germline gene expression and the brood size upon passage through dauer [13].

The compromise of the dsRNA importer *sid-1* only partially rescues the AMPK-dependent sterility of PD AMPK mutants, suggesting that the abnormal transfer of small RNAs that occurs in the absence of AMPK is registered in the germ line, culminating in PD sterility. The inability to fully restore fertility by removing *sid-1* suggests that additional Sid family genes may be involved in the systemic transfer of small RNAs from the soma to the germ line [55, 56]. Moreover, recently characterized SID-1–independent pathways might also contribute to the regulated uptake of small RNAs [57]. At present, our data cannot discern whether AMPK regulates the systemic transfer of small RNAs directly or whether it is involved in selecting the appropriate sequence-specific small RNAs to ensure the correct adjustment to gene expression is achieved during the dauer stage.

Although our data show that small RNAs and neuronal/excretory AMPK function are sufficient to mediate the proper chromatin changes in the germ cells to support a lengthy diapause, it remains unclear whether these pathways are indeed necessary to trigger these changes in the germ line. It is formally possible that the latter may proceed without any requirement for small RNAs of somatic origin or AMPK function outside the germ line. New tissue-specific approaches to protein compromise will be very important to clarify this dichotomy [58].

The stress associated with the dauer stage is documented molecularly in the chromatin modifications that govern gene expression in PD animals. How this modification in transcriptional output provides some adaptive advantage has not yet been unequivocally determined, but depending on the trigger for dauer entry [12, 49], animals that transit through dauer tend to live longer and have a significantly higher brood size, suggesting that some unforeseen complexities link the quiescence associated with dauer and subsequent brood size. These changes may be heritable, since animals that remain in dauer for extended periods also show significant alterations in their gene expression while also demonstrating an enhanced resistance to starvation in subsequent generations [55]. Therefore, the dauer larva may be a perfect example of how perceived environmental duress is transduced to the germ line. Most importantly, this may occur through a somatic sensing mechanism that could include AMPK and its ability to modulate diverse stress-specific epigenetic changes via the endogenous small RNA pathway.

At the turn of the last century, August Weismann postulated that the germ line was exclusively responsible for the heritable nature of specific traits with little or no impact from the soma [56]. Although this view is widely accepted, diverse situations have been described in which the soma acts as a critical regulator of epigenetic change with phenotypic effects that can last for multiple generations [41, 57, 58]. AMPK may be one of the critical somatic effectors required to bridge the proposed Weismann barrier, coordinating epigenetic change in the germ line with physiological or environmental cues sensed by the soma (Fig 9). Our findings therefore provide a means of dissecting the mechanisms through which the soma communicates with the germ line in order to adapt to acute environmental challenges, through the generation of a suite of chromatin modifications that are essential for poststress reproductive fitness.

## Materials and methods

### *C*. *elegans* genetics

All *C*. *elegans* strains were maintained at 15 °C and according to standard protocols [59]. The strains used for the study include CB1370 [*daf-2(e1370) III*]; MR1000 [*daf-2(e1370) aak-1(tm1944) III; aak-2(ok523) X*]; MR0480 [*daf-7(e1372) III; aak-2(ok523) X*]; MR1175 [*aak-1(tm1944) III; aak-2(ok523) X*]; MR2137 [*daf-2(e1370) aak-1(tm1944) III; aak-2(ok523) X; ltIs4[unc-119(+)Ppie1*::*plc*::*mCherry]*]; MR2156 [*daf-2(e1370); ltIs44[unc-119(+)Ppie1*::*plc*::*mCherry]*]; MR2193 [*daf-2(e1370) aak-1(tm1944) III; aak-2(ok523) X; sid-1(rr167) V*]; MR2190 [*daf-2(e1370) aak-1(tm1944) dcr-1(mg375) III; aak-2(ok523) X*]; *dcr-1(mg375)*, which carries a substitution within the helicase domain of DICER and results in defects in endogenous siRNA expression [60]; MR2191 [*daf-2(e1370) aak-1(tm1944) III; ergo-1 (tm1860) V; aak-2(ok523) X*]; *ergo-1(tm1860)*, which possesses a deletion and results in up-regulation of class II oocyte/embryo small RNAs [61]; MR1947 [*aak-1(tm1944) hrde-1(tm1200) III*, *aak-2(ok523) X*]; and *hrde-1(tm1200)*, which also has a deletion that results in a premature stop codon before the crucial PAZ and PIWI domains [62]. MR2022 [*daf-2(e1370) aak-1(tm1944) rde-4(rr1234) III*; *aak-2(ok523) X*] was generated using CRISPR, and a stop codon was inserted in exon 1 after 43 amino acids. We further characterized this allele and noted that it exhibited an RNAi-deficient phenotype similar to the previously published *rde-4(ne299)* allele [63] (S5 Fig). Transgenic lines and compound mutants were created in the laboratory using standard molecular genetic approaches. To create transgenic lines to express tissue-specific *aak-2*, MR1000 animals were injected with different constructs as per [39].

### RNAi feeding

Bacterial clones expressing dsRNA from the RNAi library were grown in LB medium with ampicillin at 37 °C overnight. The bacterial culture was seeded onto regular NGM plates containing ampicillin and IPTG (1 mM). Seeded plates were incubated at room temperature for 24 h to induce dsRNA expression. L4 larvae were fed on the RNAi plates and were allowed to lay eggs at 15 °C, and then the eggs were switched to 25 °C to induce dauer formation. For S4 Fig, increasing concentrations of IPTG were used to enhance RNAi induction and hence the degree of the RNAi effect.

### DAPI staining and counting germ cell nuclei

For whole-animal DAPI (4′,6-diamidino-2-phenylindole) staining, dauer larvae were washed off plates and soaked in Carnoy’s solution (60% ethanol, 30% acetic acid, 10% chloroform) on a shaker overnight. Animals were washed twice in 1× PBS + 0.1% Tween 20 (PBST) and stained in 0.1 mg/ml DAPI solution for 30 min. Finally, larvae were washed 4 times (20 min each) in PBST and mounted in Vectashield medium. The total number of germ cell nuclei per dauer gonad was then determined based on their position and nuclear morphology.

### Dauer recovery assay

A population of the genetically identical animals were synchronized, and the resulting embryos were added to normal NGM plates seeded with *Escherichia coli* (OP50) and incubated at 25 °C for 72 h in order to induce dauer formation and allow animals to spend at least 24 h in the dauer state. Following this window, dauer larvae were transferred to regular NGM plates and were shifted to the permissive temperature of 15 °C to allow dauer larvae to recover and initiate regular development. Upon dauer exit, the L4 larvae were individually isolated onto separate plates and were transferred to new plates at 24-h intervals to quantify their brood size. The brood size of each animal was the sum of nonhatched and hatched progeny.

For S4 Fig, animals were shifted to 15 °C but were maintained on the RNAi plates during the dauer recovery.

### Immunostaining and quantification

For extruded dauer gonad staining, gonads were dissected, fixed, and stained as described elsewhere [64]. The following primary antibodies were used: rabbit polyclonal anti-H3K4me3 (1:500; Abcam, Cambridge, UK), anti-H3K9me3 (1:500; Cell Signaling Technology, Danvers, MA, USA), and rabbit anti-HIM-3 (gift from Zetka lab, 1:200). Secondary antibodies were Alexa-Fluor-488–coupled goat anti-rabbit (1:500; Life Technologies, Carlsbad, CA, USA). Microscopy was performed as described in [65]. Ratios for the fluorescence intensity across the germ line were determined using Image J.

### Western blot

*C*. *elegans* dauer larvae and PD adults were lysed by sonication in lysis buffer (50 mM Hepes [pH 7.5], 150 mM NaCl, 10% glycerol, 1% Triton X-100, 1.5 mM MgCl_2_, 1 mM EDTA, and protease inhibitors). Protein concentrations were determined using a NanoDrop 2000C spectrophotometer (Thermo Fisher Scientific, Waltham, MA, USA). Nitrocellulose membranes were incubated with primary antibodies: rabbit anti-H3K4me3, anti-H3K9me3, anti-H3K27me3, anti-H3K9ac (1:1,000; Diagenode, Seraing, Belgium), and mouse anti-α-tubulin (1:3,000; Sigma-Aldrich, St. Louis, MO, USA). Proteins were visualized using horseradish-peroxidase–conjugated anti-rabbit or anti-mouse secondary antibody (Bio-Rad, Hercules, CA, USA).

### RNA isolation and real-time PCR

Total RNA was extracted with Trizol (Invitrogen, Carlsbad, CA, USA). RNA concentration and purity were determined with a NanoDrop 2000C spectrophotometer. Purified RNA (400 ng) was used to synthesize cDNA. Gene expression levels were determined by real-time PCR with the SYBR Green Supermix and Bio-Rad iCycler Real Time PCRSystem (Bio-Rad). Relative gene expression was normalized to *tba-1*, which was the loading control.

## Supporting information

S1 Fig AMPK acts downstream of major pathways regulating dauer entry. (A) All adult animals that laid eggs were considered as fertile. Both *daf-2 aak-1* and *daf-2; aak-2* were maintained in the dauer stage for 24 h, after which they were switched to permissive temperature to resume reproductive development. There was no significant difference on the reproductive capacity in the PD animals. (B) If AMPK mutants along with the mutations in *daf-7*/TGF-β are grown under normal conditions; they are absolutely fertile. But, upon dauer passage, *daf-7;aak(0)* animals displayed severe sterility. Similarly, *aak(0)* animals treated with dauer pheromone [*aak(0)*^*#*^] exhibited high sterility upon dauer recovery. ***P < 0.0001 using Marascuilo procedure. Assays were performed 3 times, and the data represent the mean ± SD for *n* = 50. Underlying data can be found in S1 Data. *aak*, AMP-activated Protein Kinase subunit; AMPK, AMP-activated Protein Kinase; DAF, DAuer Formation abnormal; PD, post-dauer; TGF-β, Transforming Growth Factor β.
(TIF)

S2 Fig Compromise of the small RNA pathway and the somatic expression of AAK-2 partially rescues low brood size in AMPK PD animals. (A) To assess general reproductive capability, the F_1_ progeny fertile animals were counted following the RNAi treatment under normal growing conditions (no transit through dauer). *n* = 30. (B) F_1_ progeny number were counted in the fertile animals following the dauer passage, and the total distribution is plotted. The mean brood size for each group is depicted by the horizontal red line. ***P < 0.0001, **P < 0.001, and *P < 0.05 using one-way ANOVA when compared to *daf-2;aak(0)*. *n* = 50. Underlying data can be found in S1 Data. *aak*, AMP-activated Protein Kinase subunit; AMPK, AMP-activated Protein Kinase; DAF, DAuer Formation abnormal; PD, post-dauer; RNAi, RNA interference.
(TIF)

S3 Fig Loss of *glp-1* reduces the number of germ cells in the dauer germ line. (A) *glp-1*(RNAi) was used to reduce the number of germ cells in the dauer larvae. Whole-worm DAPI staining was performed to quantify the number of germ cells, and *glp-1*(RNAi) results in significant reduction in the number of germ cells. ***P < 0.0001 using the two-tailed *t* test. *n* = 25. Underlying data can be found in S1 Data. *glp*, Germline Proliferation abnormal; RNAi, RNA interference.
(TIF)

S4 Fig Compromise of small RNA pathway components during dauer recovery fails to restore fertility. (A) To also compromise the function of the small RNA pathway during dauer recovery, *daf-2;aak(0)* animals were subjected to RNAi by dsRNA feeding against multiple components of the endogenous RNAi pathway and were allowed to recover on the RNAi plates. The L4440 empty RNAi vector was used as a control. To titrate RNAi phenotypes, increasing concentrations of IPTG were used. Fertility was significantly reduced by *dcr-1*, *rde-4*, and *ergo-1* RNAi with the increasing concentration of IPTG. *P < 0.05 using Marascuilo procedure, and *n* = 100 when compared to L4440. (B) Alleles for the *dcr-1*, *rde-4*, *ergo-1*, and *hrde-1* were introduced into the *aak(0)* background and were assessed for PD fertility to validate the RNAi results. ^#^Animals were treated with dauer pheromone to induce dauer. (C) Continuously developing animals were subjected to RNAi by dsRNA feeding against components of the small RNA pathway. ***P < 0.0001 and **P < 0.001 using Marascuilo procedure when compared to L4440, and *n* = 100. Underlying data can be found in S1 Data. *aak*, AMP-activated Protein Kinase subunit; DAF, DAuer Formation abnormal; *dcr-1*, DiCer Related 1; dsRNA, double-stranded RNA; ERGO-1, Endogenous-RNAi–deficient arGOnaute 1; *hrde-1*, Heritable RNAi Deficient 1; *rde-4*, RNAi DEfective 4; RNAi, RNA interference.
(TIF)

S5 Fig *rde-4* and *sid-1* mutants exhibit RNAi resistance. (A) To characterize the CRISPR-generated mutants, animals were subjected to *pos-1* RNAi, and viable F_1_ progeny were quantified. *n* = 20. Underlying data can be found in S1 Data. CRISPR, Clustered Regularly Interspaced Short Palindromic Repeats; *pos-1*, POSsterior localization/posterior lineage defective; *rde-4*, RNAi DEfective 4; RNAi, RNA interference; *sid-1*, Systemic RNAi Defective.
(TIF)

S1 Table Somatic defects in mutants that suppress sterility in PD AMPK mutant adults. Somatic defects were quantified in various mutant backgrounds that suppress the sterility typical of AMPK mutant PD adults. Mutation that suppress the PD sterility also partially suppress the somatic defects in the *daf-2; aak(0)* PD animals. ***P < 0.0001, **P < 0.001 and *P < 0.05 using a chi-square test. *aak*, AMP-activated Protein Kinase subunit; AMPK, AMP-activated Protein Kinase; DAF, DAuer Formation abnormal; PD, post-dauer.
(DOCX)

S2 Table The PD AMPK germ line exhibits severe architectural defects in oocyte organization and gonadal symmetry. Germline morphology was monitored in AMPK mutant PD animals using a germ cell membrane marker. *Germ lines that lacked the typical single-file organization. **An asymmetric gonad refers to a gonad with irregular gonadal symmetry in terms of size and shape of the gonadal arms. AMPK, AMP-activated Protein Kinase; PD, post-dauer.
(DOCX)

S1 Data Data underlying Figs 1–8 and S1–S5 Figs. (XLSX)

## Abbreviations

*aak*: AMP-activated Protein Kinase subunit
*ain*: Alg1 INteracting protein
AMPK: AMP Kinase
CGC: *Caenorhabditis elegans* Genetic Center
*ckb*: Choline Kinase B
*csr-1*: Chromosome-Segregation and RNAi deficient 1
DAF: DAuer Formation abnormal
*dcr-1*: DiCer Related 1
*dpy*: DumPY
dsRNA: double-stranded RNA
*elt*: Erythroid-Like Transcription factor family
ERGO-1: Endogenous-RNAi–deficient arGOnaute
FOXO: FOrkhead boX O
*glp*: abnormal Germline Proliferation
GSC: germline stem cell
H3K4me3: histone H3 lysine 4 trimethylation
H3K9ac: Histone H3 Lysine 9 Acetylation
H3K9me3: histone H3 lysine 9 trimethylation
HIM-3: High Incidence of Males 3
*hrde-1*: Heritable RNAi Deficient 1
*irld*: Insulin/EGF-Receptor L Domain protein
*ksr*: Kinase Suppressor of activated Ras
LKB1: Liver Kinase B 1
LSD1: Lysine-Specific histone Demethylase
MAPK: Mitogen Activated Protein Kinase
*mek*: MAP kinase kinase or Erk Kinase
miRNA: microRNA
MSP: Major Sperm Protein
NGM: Nematode Growth Medium
PAR-4: Partitioning Defective 4
PBST: 1× PBS + 0.1% Tween 20
PD: post-dauer
PLC: PhosphoLipase C
*pmk*: P38 Map Kinase
*Ppie*: Promoter of Pharynx and Intestine Excess
*ppk*: PIP kinase
*pro*: Proliferation of germ line
PTEN: Phosphatase and TENsin homologue
qRT-PCR: quantitative Reverse Transcription Polymerase Chain Reaction
Ras: Rat Sarcoma
*rde-4*: RNAi DEfective 4
RNAi: RNA interference
*rrf*: RNA-dependent RNA polymerase Family
*sid-1*: Systemic RNAi Defective 1
siRNA: short interfering RNA
*spe*: SPErmatogenesis
SPR: Suppressor of PResenilin defect
*srg*: Serpentine Receptor, class G 1
*sulp*: SULfate Permease family
*sur*: SUppressor of activated *let-60* Ras
*tba*: TuBulin Alpha
TGF-β: Transforming Growth Factor β
*unc*: UNCoordinated
