# Supplementary figures and images for: AMPK regulates germline stem cell quiescence and integrity through an endogenous small RNA pathway
Source: PLoS Biol. 2019 Jun 5;17(6):e3000309. doi: 10.1371/journal.pbio.3000309 (PMC6576793; doi:10.1371/journal.pbio.3000309)

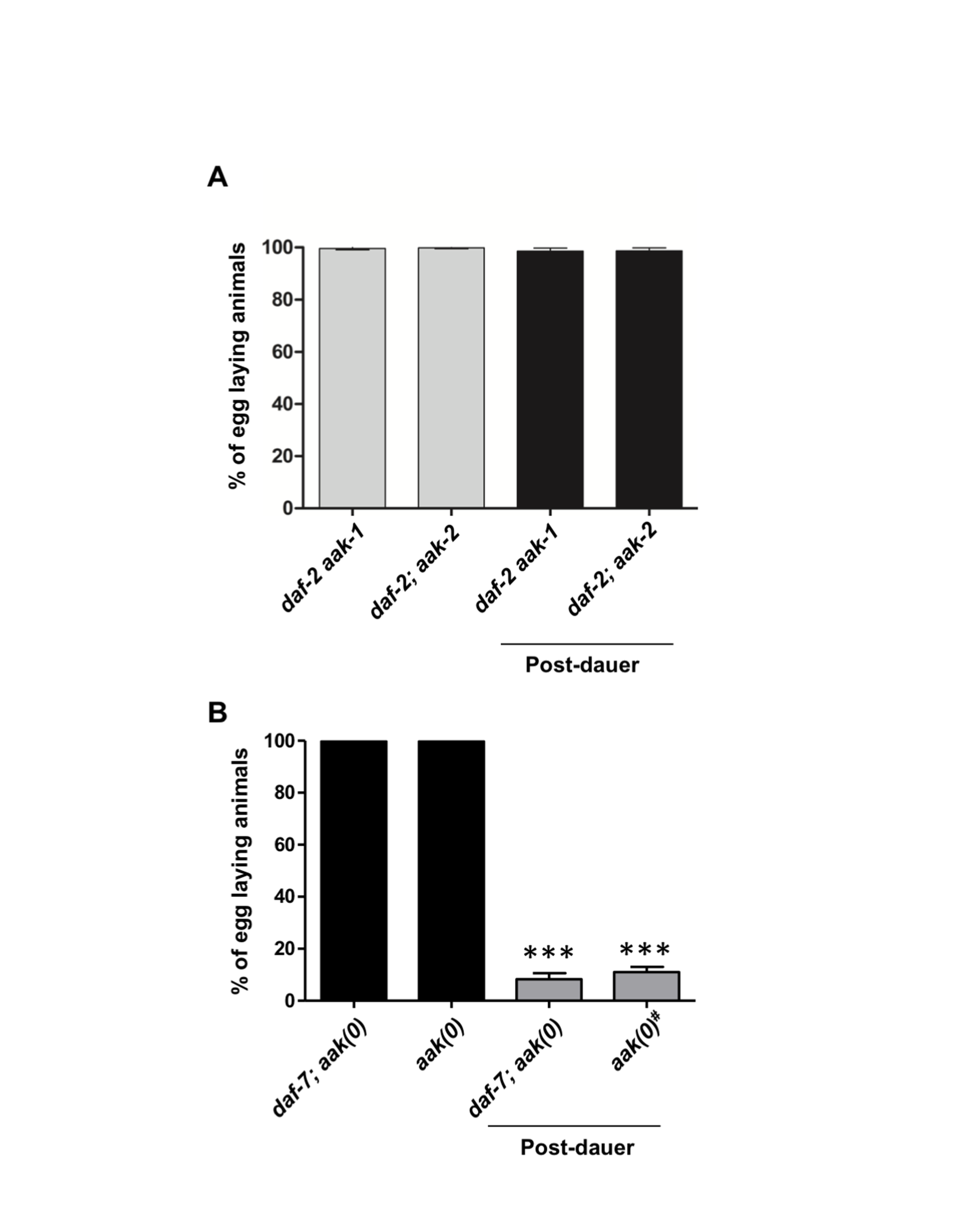

Supplement: S1 Fig — (A) All adult animals that laid eggs were considered as fertile. Both daf-2 aak-1 and daf-2; aak-2 were maintained in the dauer stage for 24 h, after which they were switched to permissive temperature to resume reproductive development. There was no significant difference on the reproductive capacity in the PD animals. (B) If AMPK mutants along with the mutations in daf-7/TGF-β are grown under normal conditions; they are absolutely fertile. But, upon dauer passage, daf-7;aak(0) animals displayed severe sterility. Similarly, aak(0) animals treated with dauer pheromone [aak(0)#] exhibited high sterility upon dauer recovery. ***P < 0.0001 using Marascuilo procedure. Assays were performed 3 times, and the data represent the mean ± SD for n = 50. Underlying data can be found in S1 Data. aak, AMP-activated Protein Kinase subunit; AMPK, AMP-activated Protein Kinase; DAF, DAuer Formation abnormal; PD, post-dauer; TGF-β, Transforming Growth Factor β. (TIF) [file pbio.3000309.s001.tif]

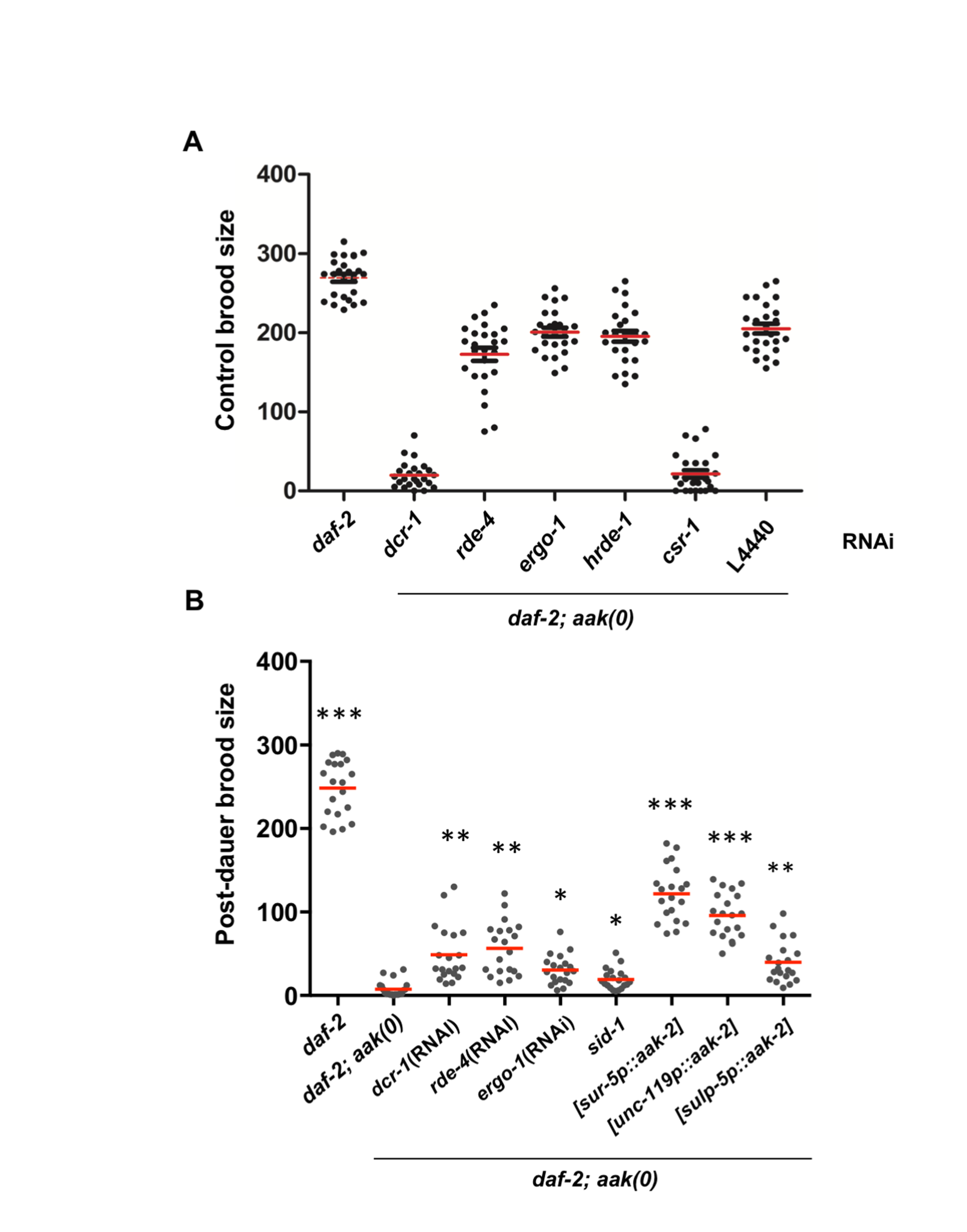

Supplement: S2 Fig — (A) To assess general reproductive capability, the F1 progeny fertile animals were counted following the RNAi treatment under normal growing conditions (no transit through dauer). n = 30. (B) F1 progeny number were counted in the fertile animals following the dauer passage, and the total distribution is plotted. The mean brood size for each group is depicted by the horizontal red line. ***P < 0.0001, **P < 0.001, and *P < 0.05 using one-way ANOVA when compared to daf-2;aak(0). n = 50. Underlying data can be found in S1 Data. aak, AMP-activated Protein Kinase subunit; AMPK, AMP-activated Protein Kinase; DAF, DAuer Formation abnormal; PD, post-dauer; RNAi, RNA interference. (TIF) [file pbio.3000309.s002.tif]

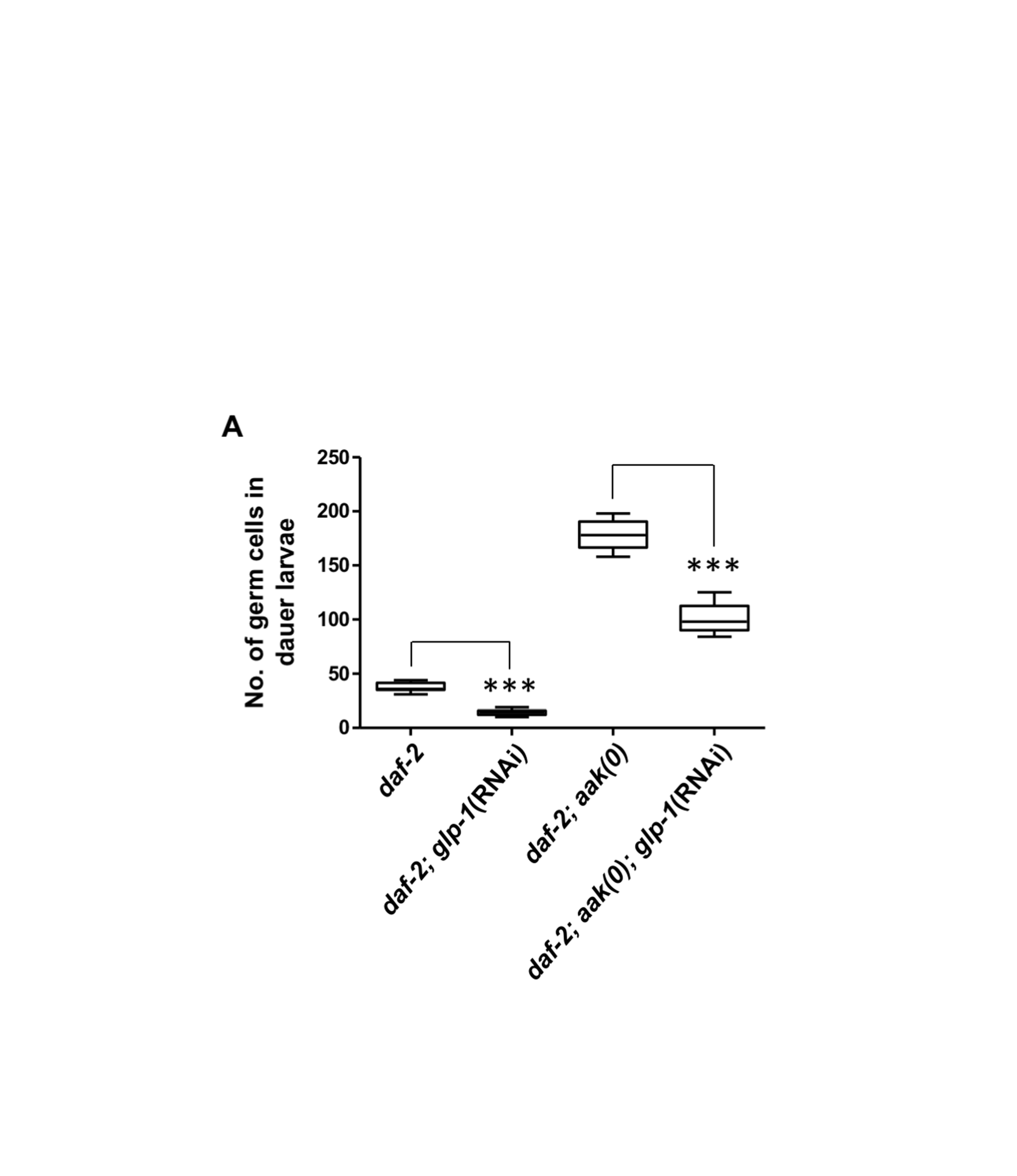

Supplement: S3 Fig — (A) glp-1(RNAi) was used to reduce the number of germ cells in the dauer larvae. Whole-worm DAPI staining was performed to quantify the number of germ cells, and glp-1(RNAi) results in significant reduction in the number of germ cells. ***P < 0.0001 using the two-tailed t test. n = 25. Underlying data can be found in S1 Data. glp, Germline Proliferation abnormal; RNAi, RNA interference. (TIF) [file pbio.3000309.s003.tif]

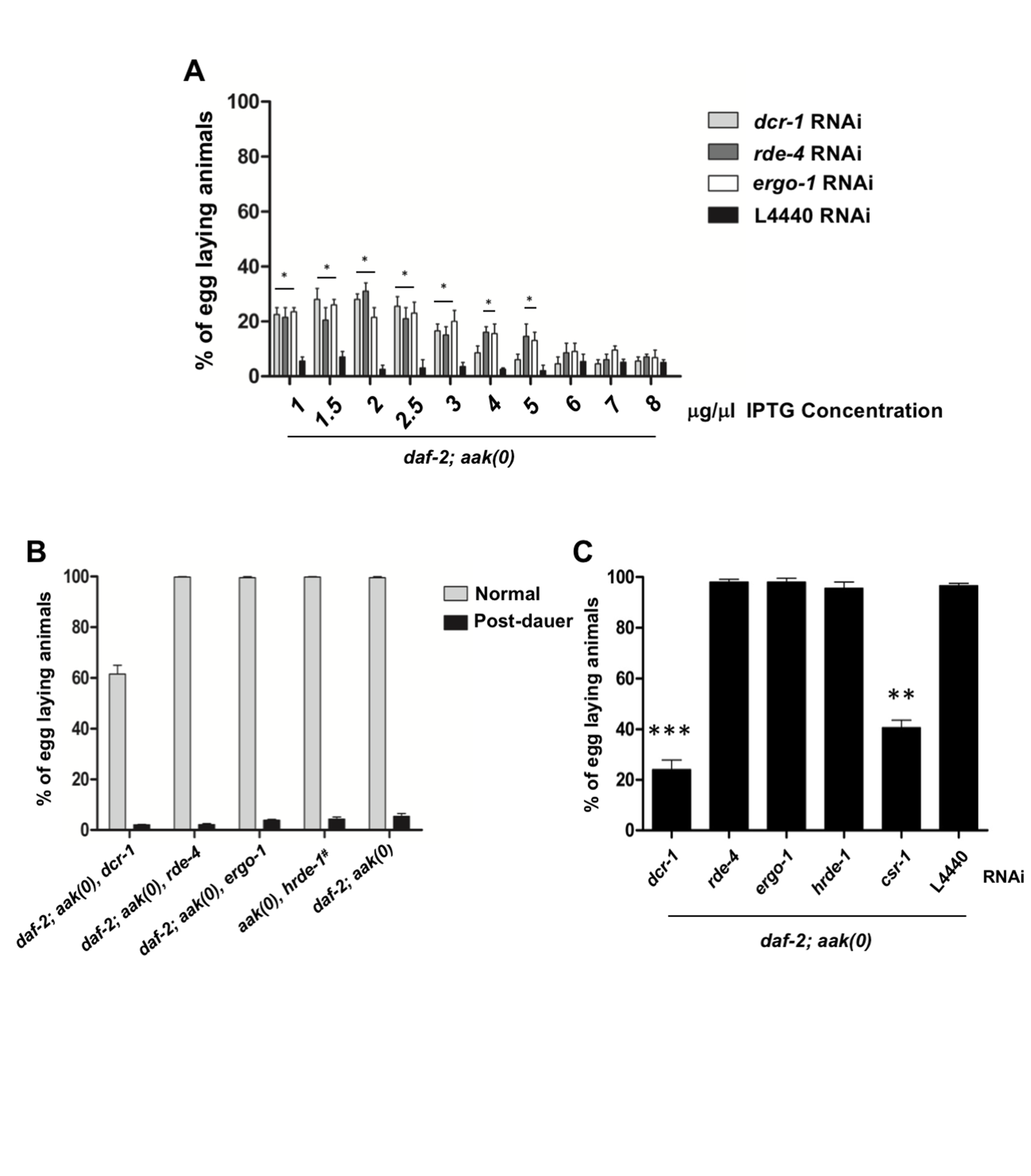

Supplement: S4 Fig — (A) To also compromise the function of the small RNA pathway during dauer recovery, daf-2;aak(0) animals were subjected to RNAi by dsRNA feeding against multiple components of the endogenous RNAi pathway and were allowed to recover on the RNAi plates. The L4440 empty RNAi vector was used as a control. To titrate RNAi phenotypes, increasing concentrations of IPTG were used. Fertility was significantly reduced by dcr-1, rde-4, and ergo-1 RNAi with the increasing concentration of IPTG. *P < 0.05 using Marascuilo procedure, and n = 100 when compared to L4440. (B) Alleles for the dcr-1, rde-4, ergo-1, and hrde-1 were introduced into the aak(0) background and were assessed for PD fertility to validate the RNAi results. #Animals were treated with dauer pheromone to induce dauer. (C) Continuously developing animals were subjected to RNAi by dsRNA feeding against components of the small RNA pathway. ***P < 0.0001 and **P < 0.001 using Marascuilo procedure when compared to L4440, and n = 100. Underlying data can be found in S1 Data. aak, AMP-activated Protein Kinase subunit; DAF, DAuer Formation abnormal; dcr-1, DiCer Related 1; dsRNA, double-stranded RNA; ERGO-1, Endogenous-RNAi–deficient arGOnaute 1; hrde-1, Heritable RNAi Deficient 1; rde-4, RNAi DEfective 4; RNAi, RNA interference. (TIF) [file pbio.3000309.s004.tif]

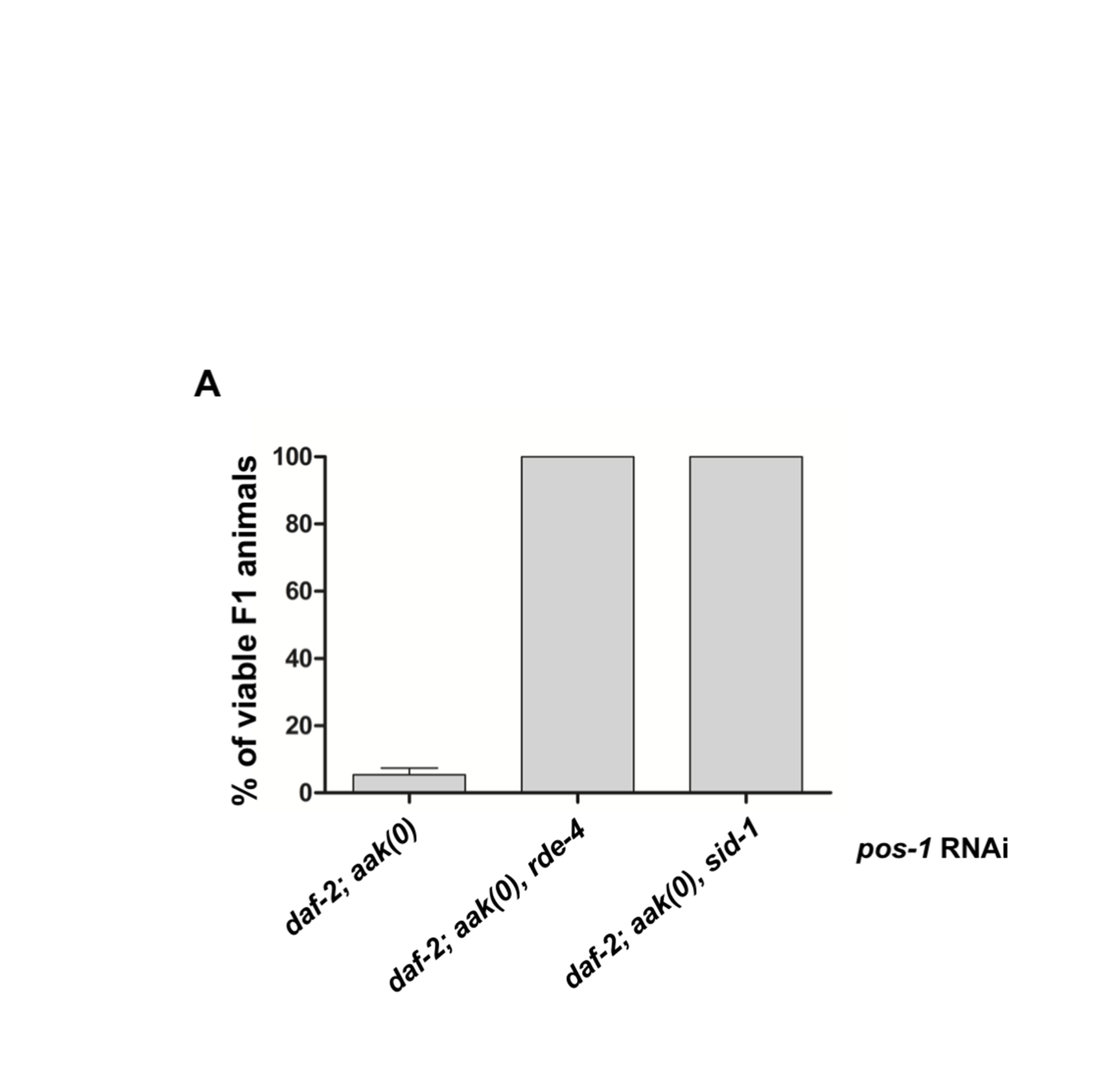

Supplement: S5 Fig — (A) To characterize the CRISPR-generated mutants, animals were subjected to pos-1 RNAi, and viable F1 progeny were quantified. n = 20. Underlying data can be found in S1 Data. CRISPR, Clustered Regularly Interspaced Short Palindromic Repeats; pos-1, POSsterior localization/posterior lineage defective; rde-4, RNAi DEfective 4; RNAi, RNA interference; sid-1, Systemic RNAi Defective. (TIF) [file pbio.3000309.s005.tif]

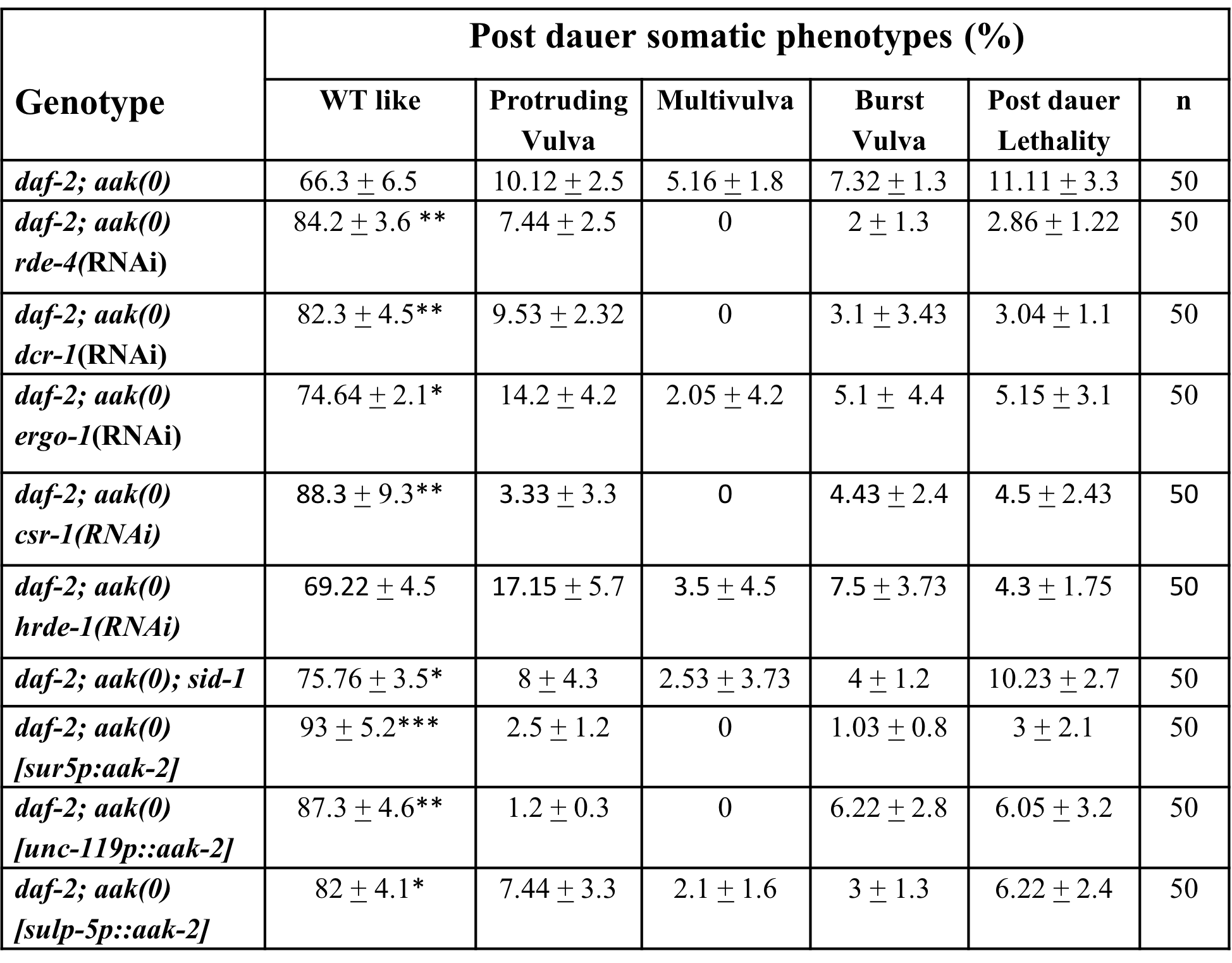


**Table S1**

Supplement: S1 Table — Somatic defects were quantified in various mutant backgrounds that suppress the sterility typical of AMPK mutant PD adults. Mutation that suppress the PD sterility also partially suppress the somatic defects in the daf-2; aak(0) PD animals. ***P < 0.0001, **P < 0.001 and *P < 0.05 using a chi-square test. aak, AMP-activated Protein Kinase subunit; AMPK, AMP-activated Protein Kinase; DAF, DAuer Formation abnormal; PD, post-dauer. (DOCX) [file pbio.3000309.s006.docx]

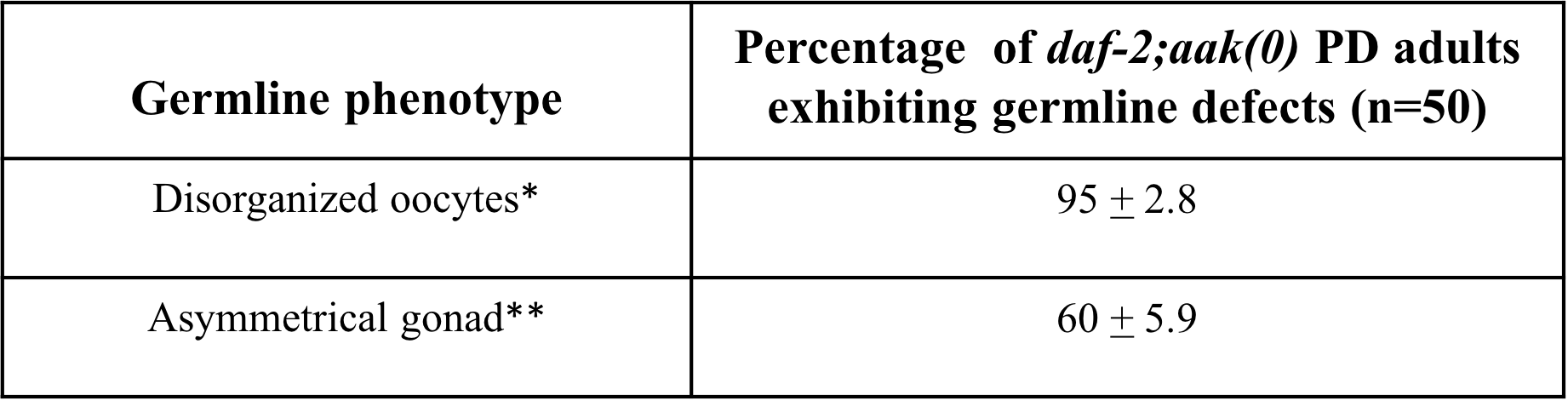


**Table S2**

Supplement: S2 Table — Germline morphology was monitored in AMPK mutant PD animals using a germ cell membrane marker. *Germ lines that lacked the typical single-file organization. **An asymmetric gonad refers to a gonad with irregular gonadal symmetry in terms of size and shape of the gonadal arms. AMPK, AMP-activated Protein Kinase; PD, post-dauer. (DOCX) [file pbio.3000309.s007.docx]
